# Sequence Divergence and Functional Specializations of the Ancient Spliceosomal SF3b: Implications in Flexibility and Adaptations of the Multi-Protein Complex

**DOI:** 10.3389/fgene.2021.747344

**Published:** 2022-01-10

**Authors:** Arangasamy Yazhini, Narayanaswamy Srinivasan, Sankaran Sandhya

**Affiliations:** ^1^ Molecular Biophysics Unit, Indian Institute of Science, Bangalore, India; ^2^ Max Planck Institute for Biophysical Chemistry, Göttingen, Germany; ^3^ Department of Biotechnology, Faculty of Life and Allied Health Sciences, M. S. Ramaiah University of Applied Sciences, Bengaluru, India

**Keywords:** SF3b, spliceosome, SF3B1, SF3B4, evolution, cancer mutations, protein-protein complexes, multi-protein assembly

## Abstract

Multi-protein assemblies are complex molecular systems that perform highly sophisticated biochemical functions in an orchestrated manner. They are subject to changes that are governed by the evolution of individual components. We performed a comparative analysis of the ancient and functionally conserved spliceosomal SF3b complex, to recognize molecular signatures that contribute to sequence divergence and functional specializations. For this, we recognized homologous sequences of individual SF3b proteins distributed across 10 supergroups of eukaryotes and identified all seven protein components of the complex in 578 eukaryotic species. Using sequence and structural analysis, we establish that proteins occurring on the surface of the SF3b complex harbor more sequence variation than the proteins that lie in the core. Further, we show through protein interface conservation patterns that the extent of conservation varies considerably between interacting partners. When we analyze phylogenetic distributions of individual components of the complex, we find that protein partners that are known to form independent subcomplexes are observed to share similar profiles, reaffirming the link between differential conservation of interface regions and their inter-dependence. When we extend our analysis to individual protein components of the complex, we find taxa-specific variability in molecular signatures of the proteins. These trends are discussed in the context of proline-rich motifs of SF3b4, functional and drug binding sites of SF3b1. Further, we report key protein-protein interactions between SF3b1 and SF3b6 whose presence is observed to be lineage-specific across eukaryotes. Together, our studies show the association of protein location within the complex and subcomplex formation patterns with the sequence conservation of SF3b proteins. In addition, our study underscores evolutionarily flexible elements that appear to confer adaptive features in individual components of the multi-protein SF3b complexes and may contribute to its functional adaptability.

## 1 Introduction

Several proteins in the cell perform vital functions as a component of specialized molecular complexes that are usually dedicated to carrying out sophisticated multistep biochemical events (Pieters et al., 2016). Like individual proteins, molecular complexes are also subject to evolutionary pressures. While evolution of single proteins has been studied extensively (Pál et al., 2006), the influence of such forces on the evolution of protein complexes is yet to be explored extensively. Advancements in cryo-electron microscopy (cryo-EM) and quantitative mass spectrometry-based proteomics, paired with affinity purification and co-immunoprecipitation, have begun to elucidate the molecular evolution of protein complexes (Hyung and Ruotolo, 2012; Stengel et al., 2012; Skiniotis and Southworth, 2016; Vimer et al., 2020) and identified distinct patterns in protein association networks between species (Wan et al., 2015). These studies have demonstrated unequivocally that protein complexes evolve through accruement of contemporary proteins, loss of primordial proteins, and modulation of protein composition and their physical connections. These phenomena are evident in multi-protein molecular machines, which perform highly complex cellular events (Marsh et al., 2013; Marsh and Teichmann, 2015; Phanse et al., 2016). A fine example of such molecular systems is the spliceosome.

The spliceosome is a eukaryote-specific molecular assembly that processes intron-containing nascent mRNA through a series of events called splicing (Collins and Penny, 2005). Intron excision by slicing and splicing of exons involves orchestrated (dis)assembly of five small ribonucleoprotein particles (U1, U2, U4, U5 and U6 snRNPs) and scores of spliceosomal proteins on to pre-mRNA. All these multi-protein/RNA complexes together form a spliceosome (Matera and Wang, 2014). The overall steps in splicing and the mechanism of two transesterification reactions are conserved among eukaryotes (Wahl et al., 2009). However, the number of protein players integrated as spliceosome in each splicing step varies remarkably from lower to higher-order eukaryotes (Jurica and Moore, 2003; Will and Lührmann, 2011). For example, pre-catalytic B spliceosome assembly has ∼110 proteins in humans while the yeast assembly contains only 60 proteins (Fabrizio et al., 2009), indicating evolutionary innovations in a selected set of eukaryotic species.

In addition to understanding the evolution of the protein complex, the observed differences in the number of players in orthologous spliceosomes attracts the question as to why such innovation occurs despite a conserved function and what adjustments ancestral components incur to achieve the changes. To address this, information on the contribution and essentiality of each component to the functions of the protein complex and interplay between components is crucial. Acquiring this information demands complementary approaches involving biochemical characterization of the function, gene manipulations, 3-D structure, sequence conservation and phylogeny of all components of the complex. Currently, such comprehensive information is unavailable for the whole spliceosome. However, SF3b, a multi-protein spliceosomal subcomplex, which functions as an integral part of U2 snRNP, has been well characterized in terms of 3-D structure and biochemical function (Das et al., 1999; Cretu et al., 2016).

The SF3b participates in both major and minor spliceosome assemblies (Golas et al., 2003). In the splicing event mediated by major spliceosomes, the SF3b helps recognize branch-point adenosine in the nascent pre-mRNA, stabilizing U2 snRNA/pre-mRNA duplex and preventing pre-mature cleavage (Will and Lührmann, 2011). The complex has seven proteins, *viz.* SF3b1, SF3b2, SF3b3, SF3b4, SF3b14b, SF3b5 and SF3b6. In yeast, the homolog of SF3b6 is absent, and hence yeast SF3b performs its function with only six components. Our recent study demonstrates that SF3b6 may play an allosteric role in the SF3b complex in a specific set of eukaryotes (Yazhini et al., 2021). This study also showed that in comparisons of yeast and human SF3b proteins, individual SF3b proteins differ substantially in length and in functional and structural domain compositions. In addition, we found significant differences in the 3-D structure and dynamics of SF3b proteins. These observations suggested considerable divergence of SF3b complex among species and invite a study on the conservation of their sequences across diverge lineages of eukaryotes.

In this study, we have undertaken a comprehensive comparative study to investigate the conservation pattern of SF3b protein components. Using a diverse set of homologs from >2000 eukaryotic species, separated by billion years of evolution (Chernikova et al., 2011), we have identified patterns of conservation and diversity in the SF3b proteins. Further, phylogenetic distribution analysis was employed to determine trends in the distribution profiles among individual protein components. These trends were then coupled with studies of multiple sequence alignments to characterize signatures of sequence divergence at the level of both the complete protein and local regions. The local regions include intermolecular interfaces, proline-rich motifs, cancer mutation sites and anti-cancer drug binding site in the individual protein components of the SF3b. Our studies show the influence of protein location within the complex and subcomplex formation patterns on the sequence conservation of SF3b proteins. The association of such patterns with taxonomic lineages reveals that *Saccharomycetales* and pathogenic protists, namely *Candida*, *Entamoeba* and *Trypanosoma* species, have diverged extensively. We find that physiochemically non-conservative residue substitutions in cancer mutation sites and anti-cancer drug binding site, as well as the lack of proline-rich motifs at the C-terminus of SF3b4, discriminate these fungi and pathogens from the rest of eukaryotes. Although the biological implications of these observations are unclear, our study unveils the signatures of sequence divergence of SF3b proteins across eukaryotes and the taxa-specific regions serving add-on functional roles that may be essential for organismal adaptation.

## 2 Materials and Methods

### 2.1 Mining Homologues of the SF3b Complex

Our study is aimed at studying conservation/divergence of functionally conserved SF3b protein complex sequences across eukaryotes. The SF3b complex is well characterized in yeast and humans and hence we considered the complex in these two species as references for this study. For the recognition of SF3b protein sequences in the entire eukaryotic domain, human and yeast SF3b proteins were considered as bait sequences and searched in the OMA database (Altenhoff et al., 2018) to retrieve orthologs. We chose to initially select orthologs because they are likely to be involved in a similar function (Koonin, 2005). Each resultant ortholog was queried, one at a time, against the OMA database to collect more distant orthologs. In addition, orthologs of each SF3b protein were collected from KEGG Orthology (Kanehisa et al., 2016) and EGGNOG databases (Huerta-Cepas et al., 2019). The use of multiple resources that are formed based on different approaches expanded the taxa sampled for ortholog identification. A union set of orthologs obtained from all three resources (one per species) was further taken as a query set and searched against the NCBI non-redundant protein sequences and the UniProt database (Uniclust30, 2018), using BLASTP (Camacho et al., 2009) and HHblits algorithms (Remmert et al., 2012) respectively. Hits from BLASTP search were parsed using an E-value threshold (0.0001) and sequence coverage threshold (70%) for both query and hits. Likewise, hits from HHblits were parsed using the E-value threshold (0.0001) and query coverage (70%) with 90% probability. Care was taken to exclude paralogs, primarily because paralogs are known to diversify in function (Koonin, 2005). We also verified that their inclusion will not significantly impact the diversity of the sequences considered for this analysis (data not shown).

Hits with less than 70% sequence coverage in both searches were further examined for the presence of functional and structural domains that are known to be associated with SF3b proteins. Domain assignment was performed using hmmscan (Eddy, 2009) against PFAM (El-Gebali et al., 2019) for functional domains and against SUPERFAMILY databases (Gough et al., 2001) for structural domains. Proteins with domain composition similar to yeast or human homolog were included in the data set. Further, domain information was used to identify potential false positives in the dataset. This filtration, using domain composition, was especially useful for multi-domain proteins *viz.* SF3b2, SF3b3 and SF3b4. Only one sequence per species was selected. This was done by mapping protein ids to species taxonomy ids through cross-referencing NCBI and UniProt databases. Subsequently, poor-quality sequences such as partial/fragment proteins, uncharacterized genomic contig sequences, proteins with segments of unknown residues and obsolete entries were discarded.

### 2.2 Multiple Sequence Alignment and Conservation Analysis

For conservation analysis, homologs were clustered at 60% sequence identity to obtain representative sequences of reasonably diverged homologs across eukaryotes, using CD-HIT (Li and Godzik, 2006). Multiple sequence alignments were performed using MAFFT-DASH algorithm with the default option for single-domain proteins (SF3b1, SF3b14b, SF3b5 and SF3b6) and E-INS-I option for multi-domain proteins (SF3b2, SF3b3 and SF3b4) (Rozewicki et al., 2019). Sequence alignment was guided by pairwise alignment of yeast and human SF3b protein structures obtained from cryo-EM structures of B^act^ spliceosome assembly, to attain reliable multiple sequence alignment (PDB codes: 5GM6 for yeast and 5Z58 for human) (Yan et al., 2016; Zhang et al., 2018). Sequences that lack functional regions (such as HEAT repeats in SF3b1 that interact with pre-mRNA/U2 snRNA as well as other SF3b proteins and two RRM domains in SF3b4) were subsequently pruned and the alignment protocol was reiterated. Alignments were manually refined to avoid gaps that interrupt protein-protein interface regions or secondary structural regions as predicted by PSIPRED method for non-human/non-yeast homologs (Buchan and Jones, 2019). Statistics of refined alignments were obtained from “alistat” and “esl-alipid” programs in HMMER package (Eddy, 1998). Conservation of each residue position was calculated using Jensen-Shannon divergence (JSD) (Capra and Singh, 2007). JSD score is an information theory-based measure that is built on the notion that the probability distribution of amino acids at residue positions evolving under “evolutionary pressure” is different from those of residue positions evolving under no pressure. It uses the BLOSUM62 matrix to derive background amino acid distribution.

### 2.3 Interface Residue Identification

To study the conservation of protein-protein interactions within the SF3b complex, interface residues were identified using protein interactions calculator or PIC (Tina et al., 2007). We used multiple available cryo-EM structures of complex A (PDB code: 6G90), pre-B (5ZWM, 6AH0 and 6QX9), B (5NRL and 5ZWO) and B^act^ (5GM6, 5Z56, 5Z57 and 5Z58) spliceosome assemblies from both human and yeast. The inclusion of multiple structures from distinct biological states that belong to different species, captures interactions that are conformation-specific and species-specific. Residues involved in hydrogen bonding and interactions with pre-mRNA and U2 snRNA were recognized using HBPLUS (McDonald and Thornton, 1994) and NUCPLOT programs (Luscombe et al., 1997). The extent of interface residue conservation was analyzed and compared among different protein-protein interfaces of the SF3b complex using the JSD score.

### 2.4 Phylogenetic Distribution of SF3b Complex

Phylogenetic profiling is a technique that infers coupling between two proteins based on the profile of joint presence/absence across a large set of species (Pellegrini et al., 1999). In addition to our homology searches in the protein databases, we probed for homologues of SF3b proteins in the genomic sequences of species covered in this study. We created a database of genome sequences of species for which assembly information is available for full genome representation or at least assembled as scaffolds. This filter was employed to consider only genomes of reasonable coverage. The details of genome availability were retrieved from the ftp site of NCBI database (https://ftp.ncbi.nlm.nih.gov/genomes/ASSEMBLY_REPORTS/, June 2021). In total, 26,123,526 genomic sequences belonging to 1838 eukaryotic species formed our nucleotide search space. For the query search, we used the reference sequence sets of recognized homologs of SF3b proteins (refer Section 2.1), clustered at 40% sequence identity. We searched our query protein sets against the prepared nucleotide database using TBLASTN algorithm (Camacho et al., 2009). The results were parsed using an E-value threshold of 10^–12^ and the query sequence coverage of at least 75%. Based on the recognition of homologs at the level of both proteins and nucleotides, we generated phylogenetic distribution profiles of all the seven SF3b proteins. The profiles were then clustered based on the presence/absence patterns using SciPy hierarchical clustering package in python programming language (Virtanen et al., 2020). In the clustering, the pairwise distance calculation was performed using “correlation” *metric* with the “average” linkage *method* to compute correlated pattern between two profiles. The heatmap figure was generated using seaborn “clustermap” function (Waskom, 2021).

## 3 Results and Discussion

### 3.1 Distribution of SF3b Homologs Across Eukaryotes

**FIGURE 1:**
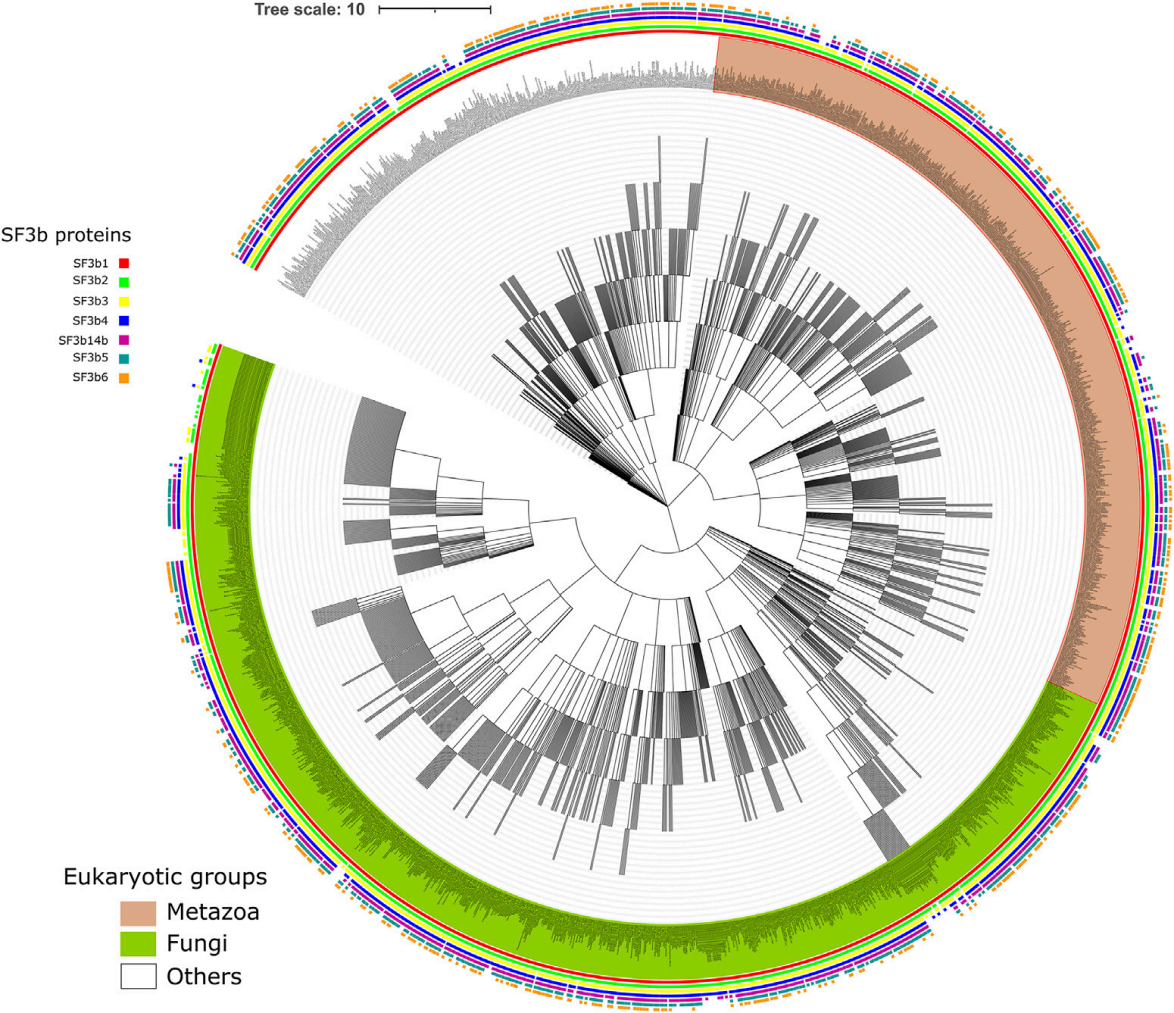
Distribution of SF3b homologs across eukaryotes. Shown is the NCBI common taxonomy tree of 2142 species that have the recognizable homolog for at least one of the seven SF3b proteins. Square boxes at the node tip in the tree indicate the presence of homologs for SF3b1 (red), SF3b2 (green), SF3b3 (yellow), SF3b4 (blue), SF3b14b (maroon), SF3b5 (dark cyan) and SF3b6 (orange). The major clades in the tree namely Metazoa and Fungi are highlighted in light brown and light green background, respectively, for their node labels. The enlarged version of the figure with legible labels can be accessed in https://doi.org/10.6084/m9.figshare.16866493.v1. The species tree was obtained from NCBI taxonomy database (Schoch et al., 2020) and the figure was generated using iTol tool (Letunic and Bork, 2021).

The ancestral SF3b complex is constituted by 7 proteins in humans and 6 in yeast. We surveyed the distribution of individual components of this complex across eukaryotes. An earlier report suggests that SF3b is likely to have been present in the last common ancestor of extant eukaryotes (Collins and Penny, 2005). Although nearly ∼1.6 million eukaryotic species are known thus far (according to the NCBI taxonomy database, June 20, 2021), the knowledge of their genome sequence is minuscule (0.6%), indicating that only limited data is available. To collect homologs from as many representative species as possible, we have employed rigorous homology searches in the known protein sequences of eukaryotic species (refer Materials and Methods). As a result, we find that 2142 eukaryotic genomes possess homologs of one or more SF3b proteins (Supplementary Table S1). Figure 1 shows the NCBI common taxonomy tree for 2124 species that we have covered in our study, with detailed representation of the distribution of SF3b homologs. Figure 1 shows that SF3b homologs are recognized in diverse eukaryotic lineages indicating that protein components are well conserved in a large variety of species. These range from microbial eukaryotes such as phytoplankton and protists to complex multicellular organisms such as humans. At the higher taxonomic level, we find that SF3b homologs are recognized across 10 major “supergroups” of eukaryotes (Supplementary Figure S1A). The species group corresponds to 1070 genera. Animals, fungi (Opisthokonta), plants (Viridiplantae) and protists (Sar) groups are the predominant members of the taxa, as seen in Figure 1. The highlighted example on the right panel of Supplementary Figure S1A illustrates that 183 SF3b homologs were recognized in 109 and 15 genera from Streptophyta and Chlorophyta clades of Viridiplantae respectively, of which 9 belong to the Oryza genus.

Furthermore, we find that homologs of all seven SF3b proteins are observed in 578 eukaryotic species. For individual SF3b proteins, the distribution shows that 1756, 1684, 1797, 1318, 1057, 1235 and 1308 species possess homologs of SF3b1, SF3b2, SF3b3, SF3b4, SF3b14b, SF3b5 and SF3b6, respectively. The comparison of species counts among SF3b proteins shows that the number of homologs that we have recognized in each of the 10 supergroups of eukaryotes is similar for all seven SF3b proteins (Supplementary Figure S1B). However, in “Opisthokonta,” we observed considerable variations in the number of homologs of each SF3b protein. We reason that the observation could be due to non-availability of data as only 52 of 1722 “Opisthokonta” species that we covered in this study have complete genome assembly information. In addition, only 20 out of the 52 completely sequence genomes have all seven SF3b proteins and were found to be human-like while 7 species had only 6 SF3b proteins and were yeast-like. Therefore, these trends are likely to change with the availability of more completely sequenced genomes. In the case of “Apusozoa,” which is a eukaryotic microbial flagellate with only limited genome sequence data available (incomplete) for only one species (*Thecamonas trahens*), we were able to recognize homologs for SF3b1 and SF3b2 proteins. Hence, we could include such a distant eukaryotic member in our analysis.

### 3.2 Components Present in the Core of the SF3b Complex are Better Conserved Than Peripheral Components

**FIGURE 2:**
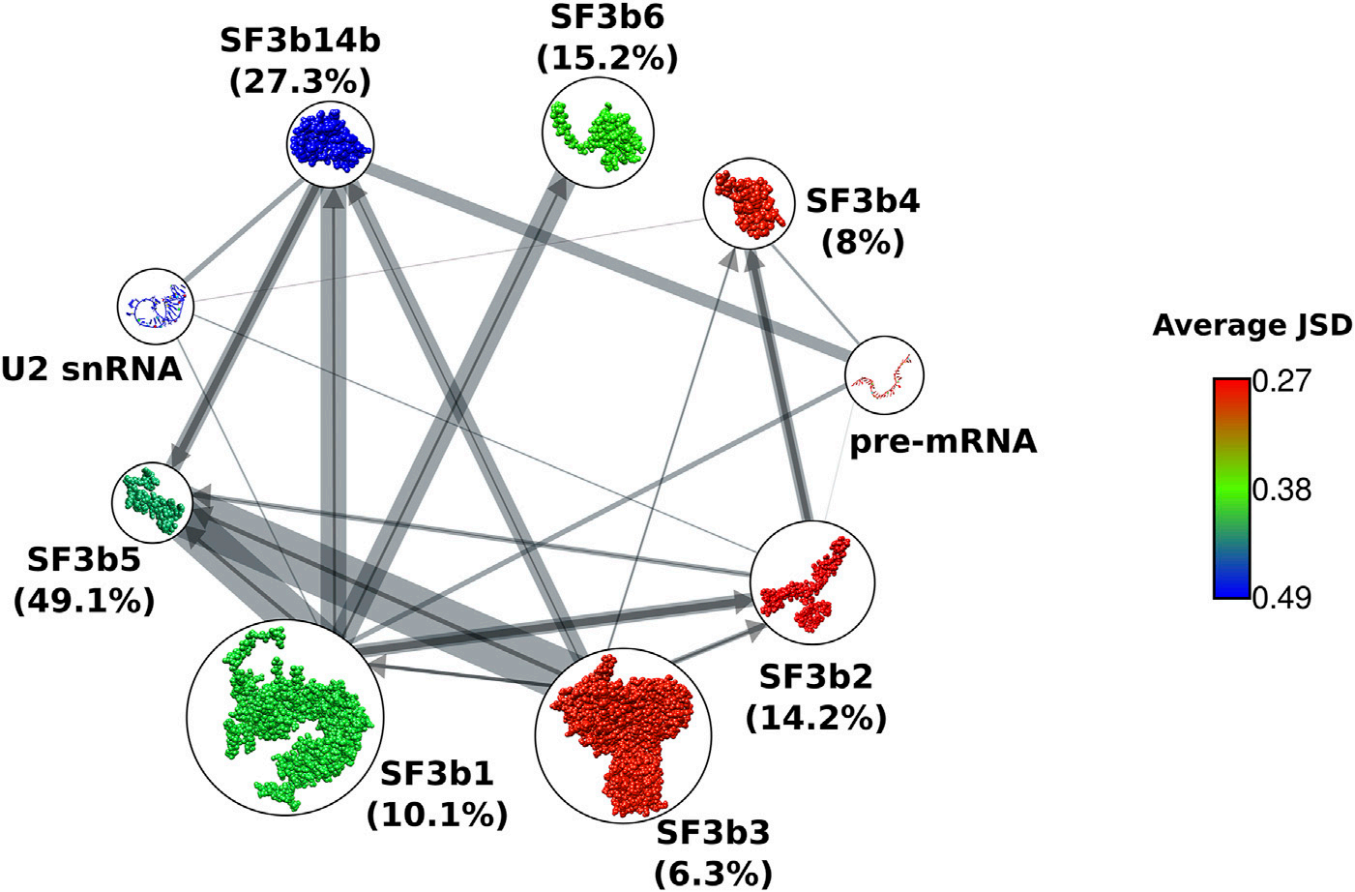
Sequence conservation of SF3b proteins. Network representation of protein-protein interactions between components of SF3b complex. Nodes show cartoon representation of SF3b proteins and RNA molecules. SF3b proteins are colored based on their average JSD score. Edge width indicates the percentage of protein sequence covered in the interface region (refer Supplementary Table S2B). To simplify representation of the bidirectional network, and an edge from a protein that shares a smaller percentage of sequence at the interface is indicated by an arrow (dark grey), while the edge from its partner having a higher percentage is indicated by a line without an arrow (light grey). Total percentage of protein sequence involved in the interface is indicated within brackets next to the node labels. Core components have higher percentages (SF3b14b and SF3b5) than the peripheral components (SF3b1, SF3b2, SF3b3, SF3b4 and SF3b6).

We then studied the overall sequence conservation using the homologues of SF3b proteins recognized in our searches. Based on the available structures of the complex, we know that in the SF3b complex, SF3b1 acts as a hub protein with the maximum number of interacting protein partners *viz.* SF3b2, SF3b3, SF3b14b, SF3b5 and SF3b6. SF3b14b and SF3b5 that reside in the interior have ∼27% and ∼49% of their residues involved in inter-component interfaces and form the core of the complex (Figure 2 and Supplementary Table S2A). The structure of the complex shows that the remaining SF3b proteins surround the core. SF3b1, SF3b2, SF3b4 and SF3b14b directly interact with pre-mRNA or U2snRNA duplex. As SF3b is an RNA interacting protein complex, the interactions with proteins and RNA molecules can both influence the evolution of individual protein components. To determine the overall sequence conservation of individual SF3b proteins, we used two sequence conservation measures based on sequence identity and conservative residue substitution patterns: 1) average pairwise sequence identity and 2) JSD score. These scores were calculated from structure-guided multiple sequence alignments of representative homologs of individual SF3b proteins, clustered at 60% sequence identity. Average pairwise sequence identity among homologs shows that SF3b1 (41%) and SF3b14b (40%) have the highest percentage of residues that remain the same across homologs. This is in agreement with their contribution to the function of the SF3b complex, as they serve to be the major components for pre-mRNA and U2 snRNA binding within the SF3b complex (Supplementary Table S2B). The same values for the other proteins such as SF3b2 (32%), SF3b3 (29%), SF3b4 (30%), SF3b5 (33%) and SF3b6 (33%) are found to be lower. Such trend is also reflected in the distribution of pairwise sequence identity among homologs. SF3b1 and SF3b14b show relatively greater number of pairs sharing above average sequence identity (Supplementary Figure S2). Whereas SF3b2, SF3b3, SF3b4, SF3b5 and SF3b6 homologs have more pairs with sequence identities below the average value.

The second conservation measure that we employed was the JSD score, where higher values indicate better conservation of residues. Here, we observe that SFb14b holds the highest average JSD score (0.49), followed by SF3b5 (0.42) and SF3b1 (0.4). SF3b6 has moderate conservation, as indicated by the average JSD score of 0.37 (Figure 2). The peripheral components SF3b2, SF3b3 and SF3b4 show lower JSD scores of 0.27, 0.28 and 0.28, respectively, among which SF3b2 and SF3b4 have RNA-binding roles. Although SF3b1 is a peripheral protein with only ∼10% of its sequence being at the interface in the context of SF3b complex, it has higher sequence conservation than the other peripheral proteins (SF3b2, SF3b3 and SF3b4) (Supplementary Table S2). When we analyze the cryo-EM structures of spliceosome assemblies, we observed that SF3b1 has ∼5% more interface residues by forming interactions with other components in the spliceosome (Supplementary Figure S3 and Supplementary Table S2C). This value is higher than the percentage of increased interface residues for SF3b2 (1.6%), SF3b3 (0.6%) and SF3b4 (2.6%) in the spliceosome assembled form. This indicates that SF3b1 acts as a core component having added interactions in the spliceosome assembled form. Hence, these additional interactions could further influence sequence evolution leading to a better conservation of SF3b1 compared to the other peripheral components of the SF3b complex.

Together, our observation suggests that the RNA-binding role results in a well conserved sequence and protein-protein interactions allow for conservative substitutions. In both scoring measures employed here, peripheral proteins show poorer conservation than the core proteins suggesting that the extent of sequence conservation is linked to the spatial location of proteins within the complex. It is especially evident in the RNA-binding peripheral components (SF3b2 and SF3b4). Together, these observations suggest that constraints due to protein-protein interactions profoundly affect the overall sequence conservation. Therefore, within a complex, proteins residing inside the complex are observed to be better conserved than the peripheral proteins. Indeed, it would be interesting to determine if similar trends are observed in other multi-protein complexes. Also, our observation is useful for inferring possible associations between proteins in the multi-protein assemblies of unknown structures and 3-D structure modeling using cryo-EM experiments.

### 3.3 Interface Residue Conservation and Phylogenetic Distribution Reveal Correlated Patterns Between Proteins Forming Subcomplexes

**FIGURE 3:**
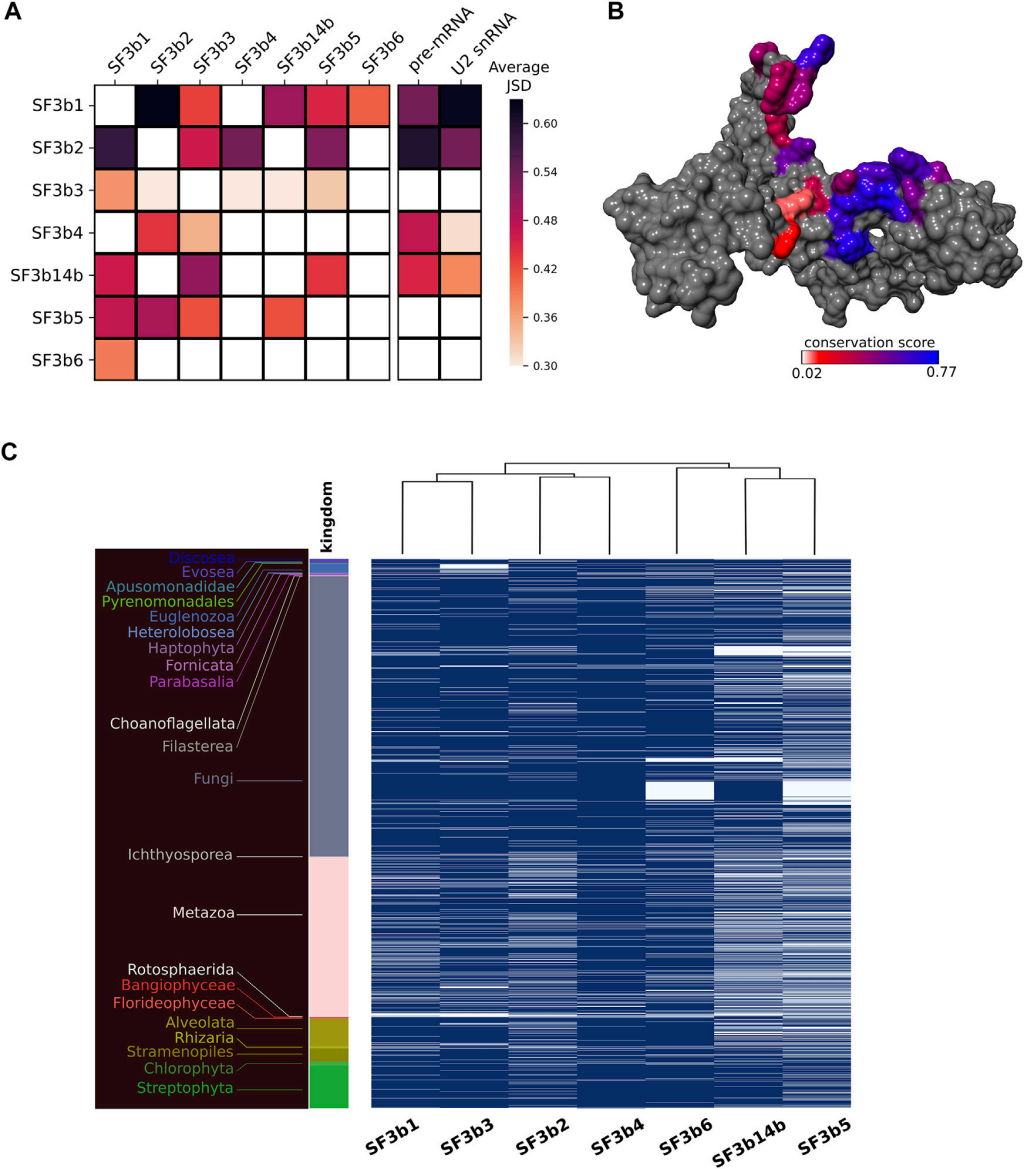
Interface conservation in the SF3b complex. **(A)** Shown is the heatmap of average JSD scores for all the observed pairwise protein-protein/RNA interfaces. Row indicates the protein to which the concerned interface region belongs and column indicates the protein partner for that interface. **(B)** Surface representation of SF3b2 with interface region for SF3b3 is colored based on conservation (JSD) score. The color gradient indicates differential conservation of residues within an interface wherein blue depicts high residue conservation while red indicates poor residue conservation. **(C)** Shown is the heatmap to represent the presence (blue)/absence (white) of each SF3b protein in the eukaryotic species. The dendrogram at the top indicates different clusters of SF3b proteins based on the similarity in their phylogenetic distribution. For example, SF3b1 and SF3b3 that share similar profiles are grouped together as SF3b14b and SF3b5. The different color bars and corresponding labels to the left indicate species belonging to different kingdoms.

Our observation of varied conservation between core and peripheral components prompted us to perform focused analysis in interface regions. In total, the SF3b complex comprises 12 protein-protein interfaces and 8 protein-RNA interfaces. Since some interactions are specific to a functional state or species, we analyzed multiple structures of humans and yeast to identify interface residues that might have otherwise been missed, if only one structure of SF3b complex structure was studied. We observed that SF3b1 and SF3b2 share the largest interface region in the SF3b complex, which involves >100 residues in the interface (Supplementary Table S2B). On the contrary, SF3b2/SF3b5 interface is the smallest, with only six residues involved. The interaction between SF3b6 and SF3b1 is mediated by 40 interface residues. The analysis of interfaces for pre-mRNA and U2 snRNA shows that SF3b1 has the largest interface regions for both RNAs. The average conservation score of interface regions reveals that SF3b1/SF3b2 interface is the most conserved interface region among the 12 protein-protein interfaces in the SF3b (Figure 3A). On the contrary, SF3b3 shows the least interface conservation, despite having a considerable number of interface residues for all its interacting partners (Supplementary Table S2B). In the case of protein-RNA interfaces, the sequence conservation varies among different RNA-binding SF3b proteins. SF3b1 and SF3b2 show high sequence conservation for the pre-mRNA interface, with a conservation score of 0.53 and 0.62 respectively than the SF3b4 (0.49) and SF3b14b (0.45). Likewise, U2 snRNA interfaces in SF3b1 (0.62) and in SF3b2 (0.55) are better conserved compared to the interfaces in the SF3b4 (0.38) and SF3b14b (0.38) (Figure 3A).

Overall, the results of interface conservation show three key observations. First, the extent of residue conservation significantly varies among different protein-protein interfaces. For instance, SF3b1 interacts with five SF3b proteins and the interface with SF3b2 is better conserved than the interfaces with other proteins, namely SF3b3, SF3b14b, SF3b5 and SF3b6 (Figure 3A). Second, a notable difference is observed in the extent of residue conservation between the interface region of two protein partners in the complex. For instance, in the SF3b3/SF3b14b interface, SF3b3 binding region in SF3b14b (JSD: 0.51) is better conserved than the SF3b14b binding region in the SF3b3 (JSD: 0.29). Third, within an interface, one part is more conserved than the other, as observed in the interface region of SF3b2 for the SF3b3 partner (Figure 3B). These observations emphasize that within a multi-protein SF3b complex, residue conservation markedly varies among different protein-protein interfaces, between interfaces of the same protein for two interacting partners and within an interface for a single partner. Interestingly, we observed that interface residues involved in bifurcated interactions with two different protein partners (overlapping interface region) are better conserved than the interface residues involved with only one protein partner (non-overlapping interface region) (Supplementary Text S1 and Supplementary Figure S4). This result supports our earlier observation of the variation in the extent of residue conservation within an interface and emphasizes that location and interactions with multiple protein partners dictate the nature of overall sequence conservation in a protein.

Furthermore, to understand the rationale for differential conservation of protein-protein interfaces of the SF3b complex and between interfaces of the same protein, we performed phylogenetic distribution analysis of the seven SF3b proteins. Typical usage of this technique is to determine correlation in the distribution profiles of proteins with the implication that proteins are functionally related show similar profiles. In the present analysis, we have adapted this technique to recognize distinct clusters between interacting partners within the SF3B complex (refer Materials and Methods section). Here, we observe that the profiles of SF3b1 and SF3b3 are similar as they clustered into a distinct group (Figure 3C). Similarly, SF3b2 and SF3b4 share similar profiles. Further, both sets jointly form a separate group from the other proteins of the SF3b complex. SF3b6 has a profile that is distinct from the cluster of SF3b14b and SF3b5. Together, these results show that SF3b1, SF3b2, SF3b3 and SF3b4 have similar profiles among themselves and that it is markedly different from the cluster formed by SF3b14b, SF3b5 and SF3b6. This observation is intriguing, especially since the SF3b14b and SF3b5 are core components of the human SF3b complex that interacts physically with SF3b1 (Golas et al., 2003; Cretu et al., 2016).

Earlier biochemical studies on the SF3b complex have demonstrated that SF3b1, SF3b2, SF3b3 and SF3b4 can form an assembly that can bind pre-mRNA (Das et al., 1999). This suggests that the assembly of these four SF3b proteins can occur independent of other SF3b components and also perform an RNA binding function. Our results on their phylogenetic distribution profiles lend support to this finding. Further, the study on individual SF3b proteins has already shown that SF3b1 and SF3b3 can associate to form a protein complex even in the absence of other SF3b proteins. Notably, our findings show that the interface conservation of SF3b3 for SF3b1 is higher than the same for other SF3b partners (Figure 3A). Likewise, it has been shown that SF3b2 and SF3b4 can interact independent of other proteins (Fromont-Racine et al., 1997; Igel et al., 1998; Das et al., 1999). We also observe that SF3b4 shows better residue conservation for the interface region with SF3b2 than the interface region for SF3b3. Therefore, our clustering results based on phylogenetic distribution analysis lend support to earlier biochemical findings that suggest that these proteins can form subcomplexes (Figure 3C). The observations point to the inherent modularity within the SF3b complex and offer clues on the nature of potential subcomplexes formed by the protein components. These results also corroborate our findings on the differential conservation of interface regions observed in the analysis of multiple sequence alignments. The lack of complete genome sequence information and inability to recognize extremely diverged homologs are factors that can influence the outcomes of such analysis. We hope that with the improvements in genome sequencing and annotation efforts more accurate estimates of such interactions may be gathered in future.

It is worth noting that SF3b proteins interact with diverse partners and play multiple roles (Sun, 2020; Yazhini et al., 2021). We show that such interactions contribute significantly to our observations on the differences in the extent of conservation at protein-protein interfaces within a complex. In addition, our earlier report has revealed that the interactions of SF3b3 with the SF3b1 vary substantially between human and yeast SF3b complexes (Yazhini et al., 2021). Similarly, the conformation of SF3b5 component differs between humans and yeast homologs within the SF3b assembled form (Yazhini et al., 2021). Collectively, these observations of differential conservation of interfaces and species-specific interaction patterns imply that the inter-protein interactions of the well conserved SF3b complex are flexible.

### 3.4 Case Studies on the Conservation Patterns of Specific Regions in the SF3b Components

We next probed into the association between the extent of conservation for proteins within the complex and their known roles in terms of biological function or disease. Below we discuss our observations in SF3b4 and SF3b1 that show taxa-specific sequence features.

#### 3.4.1 Conservation Patterns of Functional Regions in the Versatile Player SF3b4

**FIGURE 4:**
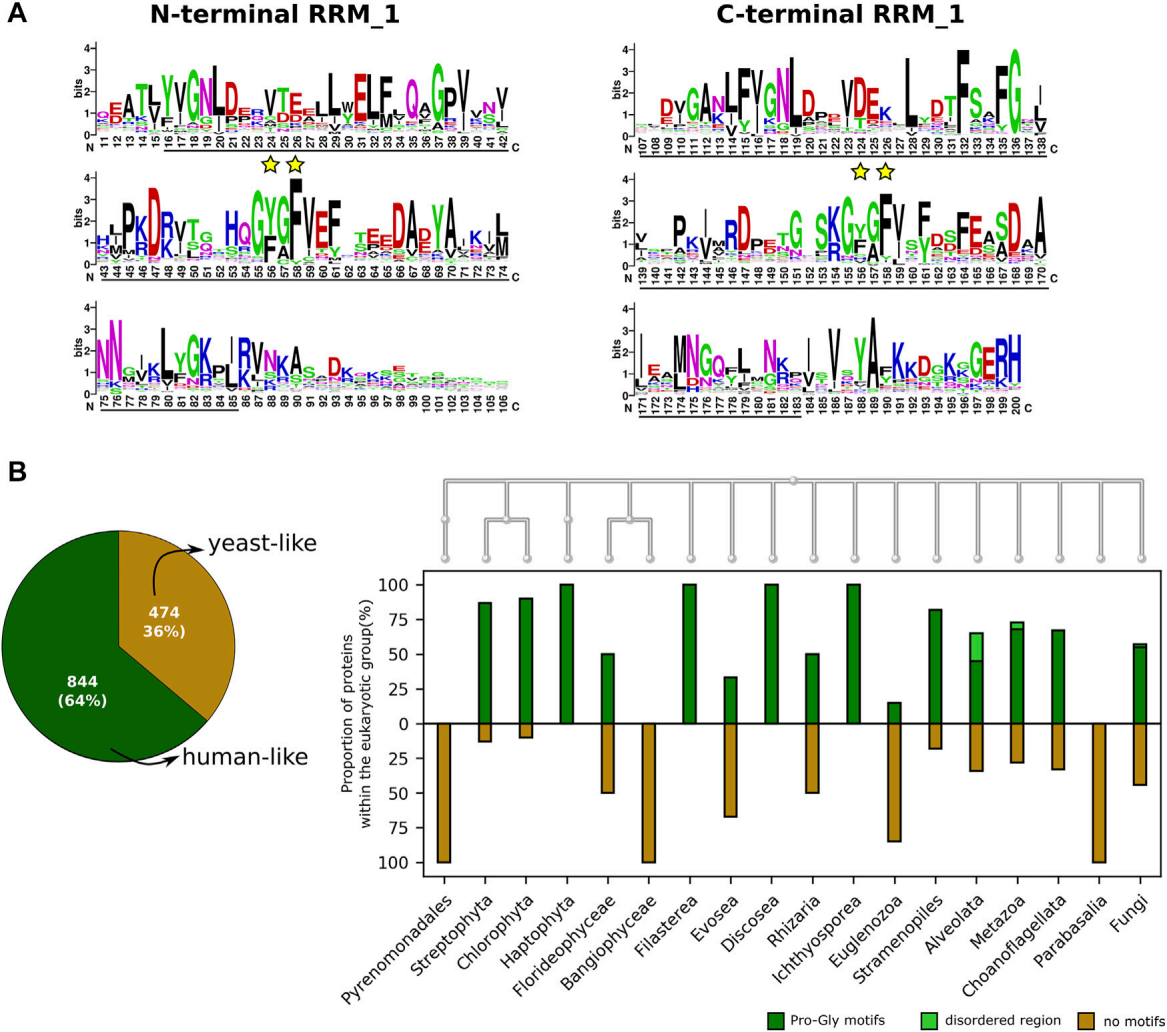
The conservation of functional sites in the SF3b4. **(A)** Shown is the sequence logo of the conservation of SF3b4 protein across eukaryotes. Black underlines highlight RRM_1 domain regions at the N- and C-terminus. Yellow stars indicate the functional sites in RNP motifs. The N- and C-terminal RRM motifs show distinct conservation profiles. **(B)** Plotted is the Venn diagram that indicates the overall percentage of homologs having recognizable motifs at the C-terminus (human-like, dark green) and homologs having no motifs at the C-terminus (yeast-like, gold). To the right, the bar plot shows the proportion of SF3b4 homologs in which the C-terminal has proline-rich motifs (dark green), disordered region (light green), or no recognizable motifs (gold). Along the *x*-axis phylogenetic links among the kingdoms having SF3b4 homologs are shown as cladogram, drawn using the NCBI Tree Viewer (https://www.ncbi.nlm.nih.gov/projects/treeview/).

SF3b4 has recently been discovered to be a versatile player. It participates in transcriptional and translational regulation of multiple genes (Watanabe et al., 2007; Ueno et al., 2019; Xiong et al., 2019) and acts as an oncogene in hepatocellular carcinoma (Iguchi et al., 2016) and as a suppressor in pancreatic cancers (Zhou et al., 2017). It comprises two RRM domains, a linker region connecting them and the C-terminal region. Based on domain assignments in all homologs, we find that both RRMs are present uniformly in all homologs as also shown elsewhere (Xiong et al., 2019). However, we observe that conservation patterns differ considerably between the two RRM domains (Figure 4A). The N-terminal RRM (average JSD: 0.47) domain is better conserved than the C-terminal RRM (average JSD: 0.4). To probe this observation at the nucleotide-level, we calculated dN/dS ratio for the two RRM domains (refer Supplementary Text S2 for method, Supplementary Table S3 and Supplementary Figure S5). Calculations based on codon substitution Model 0, a basic one ratio model (Goldman and Yang, 1994; Yang and Nielsen, 1998) show that the N-terminal RRM has the dN/dS ratio of 0.0107 while the C-terminal RRM has the value of 0.034. We further examined if the trend holds true using other codon substitution models namely Model 2a and Model 8 that allow variation in selection among sites (Nielsen and Yang, 1998; Yang et al., 2000; Yang et al., 2005). We find using Model 2a estimation that the ratios are 0.0723 and 0.1218 for N-terminal and C-terminal RRMs respectively. Likewise, the ratios are 0.0201 and 0.0452 for N- and C-terminal RRMs, respectively based on Model 8. Overall, such low dN/dS ratios of both RRM domains indicate that they evolve under evolutionary constraints. However, considerable variation (∼2 fold) between them suggests that the extent of positive selection in C-terminal RRM is relatively higher compared to the N-terminal RRM domain. It is important to note that between the two RRM motifs, the C-terminal RRM is involved in other protein-protein interactions and helps SF3b4 to perform diverse functions, independent of its role as an integral component in the SF3b complex (Watanabe et al., 2007; Ueno et al., 2019). Our observation of poor conservation in this domain implies that the amenability of C-terminal RRM to adaptive evolution may be driven by its interactions with a diverse set of proteins.

Furthermore, careful analysis of the multiple sequence alignment shows that RNP motifs, present in both RRMs that directly interact with RNA, are well conserved (Figure 4A). However, the conservation profile of key functional residues in the RNP motifs namely Tyr56 and Phe58 in the N-terminal RRM as well as Tyr156 and Phe158 in the C-terminal RRM shows that Tyr56 and Tyr156 allow considerable residue substitutions. Predominantly these involve substitutions with another hydrophobic residue phenylalanine. Also, we observed Tyr156 of C-terminal RRM is substituted by cysteine in species from 11 genera that includes *Saccharomyces* and *Candida*. This shows that these two sites are poorly conserved in comparison with Phe58 and Phe158 (highlighted in yellow star, Figure 4A). It has been shown that mutations of these two tyrosine residues impairs the RNA binding function of SF3b4 (Igel et al., 1998). Nevertheless, their poor conservation suggests that they evolve under positive selection and are evolutionarily more flexible with constraints operating on the physicochemical properties of the sites, than the other key functional residues (Phe58 and Phe158) in the RNP motifs that show conservation at the level of residue type.

Among homologs, the linker region connecting the RRMs varies from 10 to 40aa, while the C-terminal tail ranges from 1 to 477aa among homologs. This substantial variation in the length of the C-terminal tail has prompted us to study this region in detail. We find that human SF3b4 comprises proline-rich regions at the C-terminal tail. To understand their conservation, we screened all the SF3b4 homologs identified in our study (i.e., 1318 proteins) for the presence of proline-rich motifs *viz.* PPRxxP, PPPPP, PxPPxR, PPLP and PPxY in which x indicates any residue type. These motifs are reported to mediate protein-protein interactions (Ingham et al., 2005). We recognized them in the homologous sequences of SF3b4 using MAST algorithm of the MEME suite (Bailey and Gribskov, 1998). To find disordered regions in the C-terminal tail, we used InterproScan, which comprehensively integrates many protein functional sites prediction tools and maximizes the *in silico* functional characterization of proteins (Jones et al., 2014). As a result, 804 homologs were found to possess proline-rich motifs, and 40 of them comprise disordered regions. Together, we observed that 64% (844 protein) of the identified homologs possess added functional regions at the C-terminal tail akin to human SF3b4 (Figure 4B). The remaining 36% of the SF3b4 homologs (474 proteins) lack functional motifs and are similar to the yeast homolog.

When we delineated their distribution across the genomes in our dataset, we find that a considerable proportion of SF3b4 homologs have proline-rich motifs in all kingdoms except Bangiophyceae, Parabasalia and Pyrenomonadales (Figure 4B and Supplementary Table S4). Since only a few homologs were identified in these kingdoms, a conclusive inference on the implications of the absence of proline-rich motifs could not be made in these kingdoms. In the case of fungi, in which we find that yeast SF3b4 homolog (Hsh49) lacks the motifs, 55% of the identified homologs have proline-rich motifs. These observations suggest that many kingdoms of eukaryotes have a considerable proportion of SF3b4 homologs harboring proline-rich motifs as also homologs lacking such motifs (Figure 4B). Notably, we observe that the motifs are absent in SF3b4 homologs of multiple taxonomic clades, namely *Saccharomycetales*, *Trypanosoma*, *Candida*, *Streptophytina*, *Parabasalia*, as well as few metazoans (Supplementary Table S4). It is possible that these are distant homologs of the other eukaryotes with no recognizable functional features in the C-terminal tail. Further, it is possible that the SF3b4 in these species might not play versatile roles in translation and cell signalling, as observed in specific eukaryotes having SF3b4 with functional regions in the C-terminal tail (Xiong and Li, 2020). Our large-scale screening on SF3b4 homologs reveals that proline-rich motifs in SF3b4 are present in a majority of eukaryotes but selectively absent in few specific groups of pathogenic fungi, plants, protists, and parasites. This suggests that the C-terminal tail of SF3b4 is an evolutionarily flexible region and incurs taxa-specific molecular signatures. This may well be attributed to their functional adaptations and contribute to their versatility in other eukaryotes, although this remains to be experimentally demonstrated and verified.

#### 3.4.2 Residue Conservation of Key Functional Sites in the SF3b1

##### 3.4.2.1 Cancer Mutation Sites

**FIGURE 5:**
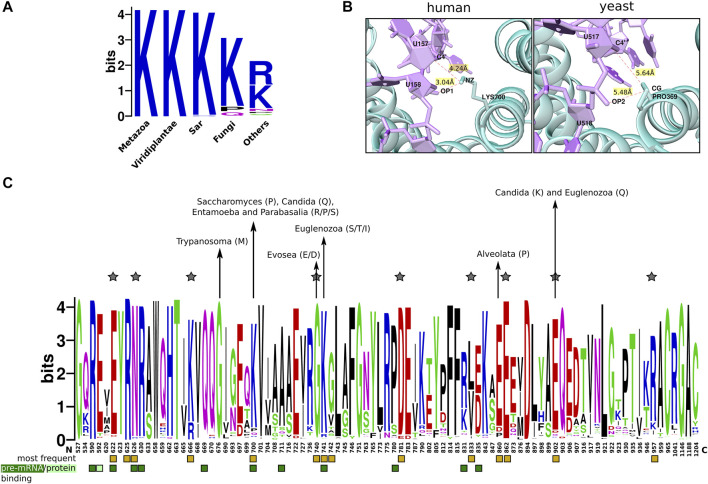
Conservation of cancer-mutation sites in SF3b1. **(A)** Sequence logo plot shows taxa-specific residue substitution of Lys700 across eukaryotes (reference is human SF3b1). **(B)** Shown is the cartoon representation of the interaction of Lys700 (or P369 in yeast homolog) with pre-mRNA in human (left) and yeast SF3b complex (right), as observed in the cryo-EM structures corresponding to PDB codes 5Z58 and 5GM6, respectively (Yan et al., 2016; Zhang et al., 2018). **(C)** Shown is a sequence logo representing residue substitution patterns of 89 cancer mutation sites (Crooks et al., 2004). Substitution patterns of residues were obtained from 1756 homologs of SF3b1. Interface residues for protein-protein interactions (light green) and protein-RNA interactions (dark green) are highlighted by square boxes at the bottom. Yellow boxes mark hotspot positions that frequently get mutated in cancers. Taxa-specific substitutions are highlighted with arrows. Positions in which the type of substitution is natural in other eukaryotes but causes cancers in humans are indicated by the star symbol.

SF3b1 is directly involved in stabilizing pre-mRNA/U2snRNA duplex. Somatic mutations in the protein are observed to be associated with several cancer conditions. Lys700Glu (or K700E) substitution is the most frequently recurring mutation across various cancers, including myelodysplastic syndromes (Seiler et al., 2018). Structural analysis shows that Lys700 physically interacts with the phosphate ion and sugar moiety of the uracil base of pre-mRNA through electrostatic interactions. Our earlier work has identified Lys700 to be a critical residue in the structural network of SF3b1 and that its perturbation affects the residue motions of the entire structure (Yazhini et al., 2021). Here, we examined residue substitution patterns among SF3b1 homologous sequences for this residue. Our analysis shows that Lys700 is fully conserved in metazoans, plants and Sar groups (protists), indicating that the residue is preserved in these eukaryotic groups (Figure 5A). In several other eukaryotic groups, we observe that the lysine has been substituted by other residues. For example, the *Saccharomyces* genus has proline, while the pathogenic *Candida* genus contains glutamine. Entamoeba and Parabasalia genus have asparagine, proline and serine residues. It is important to note from the cryo-EM structure (Yan et al., 2016) that the equivalent residue Pro369 in the yeast homolog is not involved in pre-mRNA interaction (Figure 5B). Moreover, a previous biochemical study has shown that Lys700Glu substitution does not interfere with the affinity for RNA binding or have any effect on SF3b1 interaction with other proteins (e.g., U2AF65) (Cretu et al., 2016). This observation suggests that lysine is selected mainly in the three supergroups of eukaryotes and its selectivity in these taxa is perhaps enforced by a role in pre-mRNA binding. On the contrary, genus-specific substitution in other supergroups, which are predominantly pathogens, hints that the position is susceptible to adaptive evolutionary force and might not have been selected for pre-mRNA binding, as evident in the yeast complex structure. Thus, our finding reveals and highlights taxa-specific residue substitutions at lysine 700.

We also extended our analysis to screen a comprehensive list of sites that were reported to be associated with cancers in humans. 89 residue positions are observed to be mutated in one or many cancer conditions (Cretu et al., 2016; Seiler et al., 2018). Cancer-causing substitutions at these hotspot sites is associated with aberrant splicing patterns (Dziembowski et al., 2004; Alsafadi et al., 2016). We analyzed the multiple-sequence alignments of the protein at these positions and find that they are predominantly located in the HEAT repeats 4–12. When we compute the JSD score, we find that 79% of these 89 sites have a score above 0.4, suggesting that the sites that undergo mutation in various cancers are conserved (Supplementary Figure S6A). From the structures of human and yeast SF3b complexes, we find that 11 of these mutation sites are involved in pre-mRNA and/or U2 snRNA binding (green boxes highlighted at the bottom, Figure 5C). Two other residues *viz.* Glu592 and Cys1204 are engaged in protein-protein interactions of SF3b1 with SF3b14b and SF3b3, respectively. This shows that mutations at such sites may affect SF3b1 interactions with RNAs and other SF3b components. Further, we find that in 9 sites which are located in the helical regions of HEAT repeats, residue substitutions that cause cancers in humans are naturally observed in SF3b1 homologs in other eukaryotes (grey star symbols, Figure 5C). Notably, of these 9 sites, Asn626 functions to interact with pre-mRNA. Therefore, cancer-causing residue substitutions might have influence on the pre-mRNA binding in species having such substitutions. Interestingly, when cancer-causing mutations of some of these sites (Leu822, Glu862, Glu902 and Arg957) are introduced experimentally in yeast cells, they do not show any growth defect (Kaur et al., 2020). Therefore, the observed substitution patterns suggest that although specific residue types at these positions are critical in human SF3b1 and their mutations may lead to cancers, we observe that they are not uniformly selected across eukaryotes.

Furthermore, we analyzed the conservation of these sites in 490 metazoans covered in our dataset, to understand how well these sites are conserved in closely related species of humans. We observe that 40 residues are highly conserved (JSD >0.7), of which Arg590, Gln669, Gly676, Arg775, Asp781, Glu862 and Gly1146 have no substitutions in the metazoan homologs (Supplementary Figure S6B). Among these 40 residues, six residues interact with pre-mRNA and Glu592 is involved in protein-protein interactions with SF3b14b. Interestingly, when we compare these results with the overall conservation pattern across eukaryotes, we find that a pre-mRNA binding residue Glu622, showing cancer-causing substitution Glu622Asp in humans, harbours the same substitution in the SF3b1 homologs present in a few clades. These includes species from *Brettanomyces*, *Ophiocordyceps*, *Tolypocladium* and *Zygosaccharomyces* of “Fungi” clade and *Paramecium* of “Alveolata” clade (Glu622Asp) (Supplementary Table S5). Likewise, another pre-mRNA binding residue, Asn626 possessing a cancer-causing substitution Asn626Asp in humans, shows the same substituion in two species (*Tortispora caseinolytica* and *Thecamonas trahens*). The observed trend suggests that these sites that are universally conserved in metazoans and play pre-mRNA binding roles are not well preserved in specific groups of species in other taxonomic clades and flexible enough to allow radical residue substitutions. Given that cancer-causing substitutions at these sites lead to alternative splice site selection (cryptic 5′ and 3′ splice sites) and result in defective or alternative splice variants (Darman et al., 2015; Alsafadi et al., 2016; Shiozawa et al., 2018; Liu et al., 2020), our observation of taxa-specific substitution patterns invites a detailed investigation on the link between the nature of residue type at these sites and splicing pattern. We anticipate that such a study will unravel a regulatory mechanism of gene expression mediated by splice site selection (Cooper and Mattox, 1997).

##### 3.4.2.2 Pathogenic Parasites Have Unique Residue Features in the Anti-Cancer Drug Binding Site of SF3b1

**FIGURE 6:**
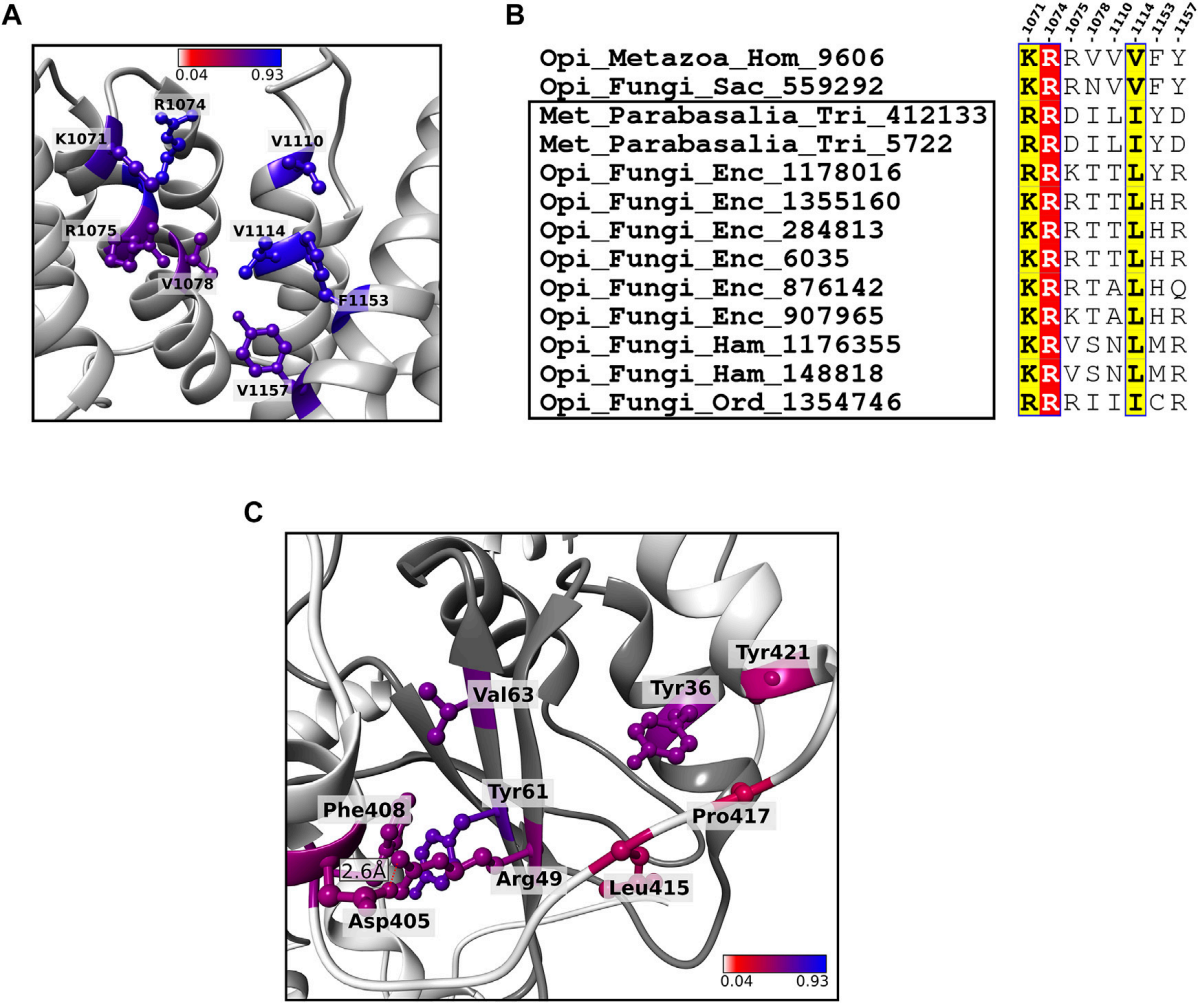
Residue conservation of key functional residues in SF3b1 and SF3b6. **(A)** Shown is the cartoon representation of SF3b1 focused at known anti-cancer drug binding site. The 8 drug binding sites are highlighted in a ball and stick representation and colored based on the conservation score. **(B)** Sequence alignment of the 8 anti-cancer drug binding sites in the SF3b1 from humans, yeast and 11 pathogenic species (highlighted within the black box) in which physicochemically non-synonymous residue substitutions are observed. The well conserved sites are highlighted by red and yellow background as per ESPript format (Robert and Gouet, 2014). **(C)** The cartoon representation shows SF3b1 (light grey) and SF3b6 (dark grey) in the complexed form. Shown in the ball and stick representation are core interface residues proposed as critical residues that stabilize the SF3b1/SF3b6 complex. Residues are recognized based on the conservation pattern and contributions to energy and the geometry of the complex. The color code of these residues is the same as in (A). Hydrogen bond formed at the core interface is shown by the red dashed line.

As SF3b1 mutations are associated with cancers, SF3b1 has been used as a target for anti-cancer therapy. Thus far, a few splicing modulators, namely Spliceostatin A, Pladienolide B and Herboxidiene have been designed against SF3b1 (Cretu et al., 2018). These drugs occlude pre-mRNA binding site and stymie SF3b1 interaction with the branch site sequence. In addition, they hamper the conformational transition of the “open” to “close” state required for SF3b1 to assemble into the spliceosome. Conservation of drug binding residues located in HEAT repeat domains 15–16 indicates that all of them are well conserved. They all have a JSD score above 0.5 in homologs across 10 eukaryotic supergroups (Figure 6A). Of the 8 residues at the binding site, three positions (Lys1071, Arg1074 and Val1078) are well preserved across eukaryotes. Of these, Lys1071 and Val1078 are directly involved in pre-mRNA binding. In addition, the mutation of Arg1074 (Arg1074His) which is present in the helical region of 14th HEAT repeat, was observed to show in phenotypic resistance for anti-cancer drug treatment (Yokoi et al., 2011; Cretu et al., 2018). This suggests that the residue potentially aids in pre-mRNA binding of neighbouring residues (His1069-Lys1071, Arg1075 and Val1078). Our observation on the absolute conservation of Arg1074 across all eukaryotes establishes its critical role in anti-cancer drug binding. In the remaining binding sites, we observed physicochemically non-conservative residue substitutions exclusively in two “Metamonada” parasites and nine fungal pathogens. For example, Val1078 is substituted by asparagine in yeast and the same position has other polar residues in selected fungal pathogens (Figure 6B). Our observation of non-conservative residue substitutions in pathogenic parasites indicates that the SF3b1 of these pathogens is distinct from human SF3b1 at these sites. Although the exact physiological implications are unclear and beyond the scope of the current study, we believe that such observations will be useful and can be exploited to appropriately modify and repurpose existing drugs, to selectively target SF3b1 proteins of such fungal pathogens and treat infectious diseases caused by them. Such findings gain significance since SF3b1 is currently being considered as an effective drug target (Bonnal et al., 2012).

##### 3.4.2.3 Identification of the Most Critical interactions for SF3b6 Association With SF3b1

Previous studies on protein interfaces defined two categories of regions within an interface: 1) “core” wherein the surface exposed residues become highly buried, i.e., relative solvent accessibility ≤0.7% upon complex formation and 2) “rim” covering the rest of the interface with residues having slightly higher solvent accessibility (between 7 and 10%) in the complexed form. The core region is indispensable for protein-protein interactions and generally, its conservation is higher than that of the “rim” region (Chakrabarti and Janin, 2002; Guharoy and Chakrabarti, 2005). In the SF3b complex, SF3b6 component is not well conserved across eukaryotes (Yazhini et al., 2021). To study the extent of conservation of interactions formed between SF3b6 component and the SF3b complex, we analyzed conservation pattern of interface residues and identified core interface residues essential for the SF3b6 association with the complex. The SF3b6 interacts solely with SF3b1 in the SF3b complex. Our analysis on multiple sequence alignments of SF3b6 homologs reveals that 5 out of 19 interface residues are highly conserved (average JSD: 0.56 and Supplementary Figure S7). The conservation score of corresponding interface residues in the SF3b1 shows an average JSD value of 0.48 (Supplementary Table S6). To predict core interface residues that form critical interactions between SF3b1 and SF3b6, we probed for a complementary conservation pattern at the interface regions between SF3b1 and SF3b6. For this purpose, we considered a residue pair to lie in the core only when one partner has JSD score >0.5 and the other partner residue has the JSD score of at least 0.4. Based on this criterion, we identified 9 residues in total *viz.* Asp405, Phe408, Leu415, Pro417 and Tyr421 from SF3b1 and Try36, Arg49, Tyr61 and Val63 from SF3b6 as the most critical interface residues. When we analyzed the available structures, we note that association between these residues is contributed by four hydrophobic interactions, one ionic and one side-chain and main-chain non-bonded interactions (Figure 6C and Supplementary Table S6A). In addition, by using PPCheck and KFC2 servers that employ energy-based and geometry-based principles, respectively, for hotspot interface residues prediction (Zhu and Mitchell, 2011; Sukhwal and Sowdhamini, 2013), we recognized that Phe408 and Leu415 in SF3b1 and all the four residues in the SF3b6 are hotspots for stabilizing SF3b1/SF3b6 interactions (Supplementary Table S6B). The conserved residues that we report in our study are observed to confer essential interactions for SF3b1/SF3b6 binding and any perturbations to them potentially impede their association. In our earlier study based on structural features and dynamics of the SF3b complex, we predict that SF3b6 is a potential allosteric regulator of the SF3b1 (Yazhini et al., 2021). We anticipate that our current funding will help in the design of *in vitro* mutagenesis experiments, to study the biological significance of SF3b1/SF3b6 association and validate our hypothesis of SF3b6 mediated allosteric regulations in pre-mRNA splicing. We believe that these observations will also be relevant to the other eukaryotic species in which SF3b6 is observed.

## 4 Conclusion

The growth of biochemical and 3-D structural data on macromolecular complexes is accelerating with the advent of large-scale proteomics and cryo-EM techniques. These data form the basis to characterize molecular complexes apropos of the nature of components, their topology, architecture etc (Marsh et al., 2015; Marsh and Teichmann, 2015). Concomitantly, there is considerable interest in the evolutionary aspects of molecular complexes, to understand how evolutionary force brings about new functions and sophisticated regulatory mechanisms in protein complexes of higher-order organisms. Studies have shown that at the coarse level, a complex evolves through rewiring of intermolecular association within the complex and addition or loss of components (Wan et al., 2015). In this context, our work provides insights on the evolution of a molecular complex by showcasing diversity in the sequence of each component among their homologs and the biological links associated with its sequence diversity in the ancient spliceosomal SF3b complex. Our findings reveal that the location and the formation of subcomplexes can have a strong influence on the sequence conservation of individual protein components. Further, their demography across eukaryotes, residue conservation patterns of key functional sites collectively showcase the greater divergence of fungal species. Specifically, species belonging to *Saccharomyces* and pathogens infecting humans from the *Candida*, *Entamoeba* and *Trypanosoma* genus are observed to have diverged extensively. We foresee that our results have potential applications in the 1) accurate structure modeling of multi-protein complexes and assemblies of such complexes from various species, 2) functional characterization of protein-protein associations between SF3b proteins through genetic manipulations and 3) detailed investigations on the role of the unique sequence signatures in the SF3b proteins of the pathogens that we have reported here.

## Data Availability

The original contributions presented in the study are included in the article/Supplementary Material, further inquiries can be directed to the corresponding authors.
