# Supplementary material for: Sequence Divergence and Functional Specializations of the Ancient Spliceosomal SF3b: Implications in Flexibility and Adaptations of the Multi-Protein Complex
Source: Front Genet. 2022 Jan 10;12:747344. doi: 10.3389/fgene.2021.747344 (PMC8785561; doi:10.3389/fgene.2021.747344)
Supplement: Supplementary file 4 [file DataSheet1.PDF]

Supplementary Information

Sequence divergence and functional specializations of the ancient spliceosomal SF3b: implications in flexibility and adaptations of the multi-protein complex **

Arangasamy Yazhini[1,2], Narayanaswamy Srinivasan[1]* and Sankaran Sandhya[1,3]*

Author Affiliations

[1]Molecular Biophysics Unit; Indian Institute of Science; Bangalore, Karnataka, 560012; India.

*To whom correspondence should be addressed.

Tel: +91 80 22932837; Fax: +91 80 23600535; Email: sandhya.bt.ls@msruas.ac.in and ns@iisc.ac.in

Current address

[2]Max Planck Institute for Biophysical Chemistry; Göttingen, Germany, 37077; yazhini@mpibpc.mpg.de

[3] Department of Biotechnology, Faculty of Life and Allied Health Sciences, MS Ramaiah University of Applied Sciences, Bengaluru 560054, India;

** This article is dedicated to one of the authors, Prof. N. Srinivasan, who passed away on September 3rd, 2021.

Supplementary Text

S1: Residues in the overlapping interfaces tend to be better conserved than residues in the non-overlapping interfaces

While analyzing the conservation of inter-component interfaces, we observed differential conservation between residues of the same interface region. Interestingly, we find that within an interface, some residues interact with more than one protein partner and form overlapping interfaces. For example, Arg181 from the yeast homolog of SF3b3 forms hydrogen bonding with both Asp84 of SF3b14b and Tyr18 of SF3b5 (Figure S3A). Likewise, within the SF3b complex, the SF3b1, SF3b2, SF3b3, SF3b14b and SF3b5 components have interface residues involved in such bifurcated interactions. We examined the extent of conservation in such overlapping interface residues and compared it with residues in the non-overlapping interface. The distribution of the JSD score (Figure S3B) shows that overlapping interface residues tend to have higher conservation than the non-overlapping interface residues in three out of five proteins that we have analyzed. SF3b1 and SF3b2 proteins are two exceptional cases and we reason that this could be due to skewed data *i.e.,* the presence of many residues at the non-overlapping interface compared to overlapping interface residues. Also, it is worth noting that a few points having low JSD scores in the SF3b2 interface correspond to poorly conserved regions due to insertions (at positions 210-221, 256-262 and 282-287) at the interface of yeast SF3b2. Nevertheless, the observation of higher conservation at overlapping interfaces than the non-overlapping interfaces in the multi-protein SF3b complex encourages a large-scale analysis. By doing so, it can be further demonstrated with statistical confidence whether the same trend holds true for other multi-protein complexes/assemblies as well.

S2: The calculation of dN/dS ratio for RRM domains in the SF3b4

To compare the rate of positive selection in N- and C-terminal RRM domains of SF3b4, we selected one representative sequence for each of the 17 different kingdoms from which we could recognize at least one SF3b4 homolog (Supplementary Table S3). We aligned those 17 representative protein sequences using MAFFT multiple sequence alignment algorithm (Rozewicki et al., 2019). We selected the regions corresponding to N-terminal RRM and C-terminal RRM domains with respect to human protein domain boundaries (UniProt entry: Q15427) using AliView software (Larsson, 2014). These two domain alignments were then used to estimate phylogenetic trees based on the maximum likelihood method using IQ-tree

algorithm (Nguyen et al., 2015). The best protein models were selected by ModelFinder (Kalyaanamoorthy et al., 2017). The trees were generated from 1000 replicates for the ultrafast bootstrap approximation as well as SH-like approximate likelihood ratio test. The alignments and phylogenetic trees are given in Supplementary Figure S5. Further, we mapped protein identifiers to mRNA sequences one by one by searching in the NCBI (https://www.ncbi.nlm.nih.gov/protein/) and UniProt (https://www.uniprot.org) databases. The nucleotide sequences and protein multiple sequence alignments were given into PAL2NAL online server to obtain the corresponding coding DNA sequences or CDS (Suyama et al., 2006). Finally, the CDS sequences and phylogenetic trees were given as inputs to CODEML program in PAML package (Yang, 1997) to calculate dN/dS ratio. We obtained the dN/dS ratios based on Model 0 (NSsites=0, Model 2a (NSsites=2) and M8 (NSsites=8). Model 0 is a basic one ratio codon substitution model and is used to calculate dN/dS ratio under natural selection (Goldman and Yang, 1994; Yang and Nielsen, 1998). Model 2a is a site-specific substitution model that allows the ratio to change among sites and generally used for the ratio estimation under positive selection (Nielsen and Yang, 1998; Yang et al., 2005). Model 8 is also a site-specific substitution model and it implements Bayes empirical Bayes (BEB) to identify sites that are under positive selection (Yang et al., 2000, 2005). We used the results from these three models and compared the estimated dN/dS ratios between N- and -C terminal RRMs of the SF3b4 to study their extent of sequence evolution at the nucleotide level.

Supplementary figures and legends

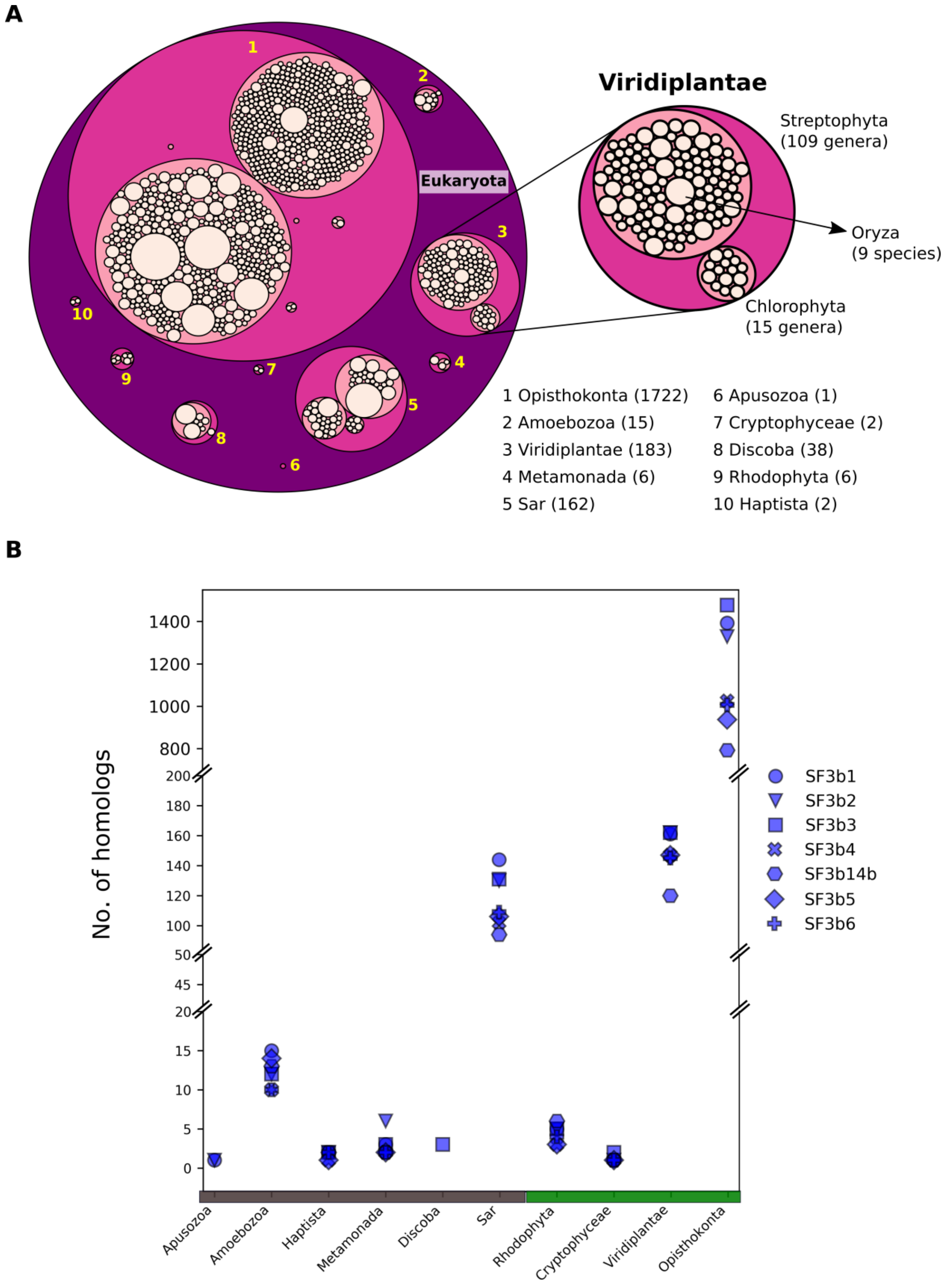

Figure S1. The distribution of homologs of seven SF3b proteins in the 10 supergroups of eukaryotes. A) The circular diagram depicts the number of eukaryotic species covered in our study. Outer to inner circles represent three hierarchical taxonomy levels: domain (i.e.,

Eukaryota), kingdoms and genus, respectively. Circle size indicates the number of species covered at each level. Numbers adjacent to primary inner circles represent 10 major eukaryotic supergroups: 1- Opisthokonta; 2- Amoebozoa; 3- Viridiplantae; 4- Metamonada; 5- Sar; 6- Apusozoa; 7- Cryptophyceae; 8- Discoba; 9- Rhodophyta; 10- Haptista. The total species covered in each supergroup is given within a bracket. A zoomed inset in the right panel exemplifies the dataset for Viridiplantae which shows that 109 and 15 genera from Streptophyta and Chlorophyta kingdoms have been covered, respectively. The size of inner light pink circles indicates the number of species belonging to each genus, as indicated by the arrow for 'Oryza' in which 9 species are covered. B) Shown as a dot plot is the total number of SF3b homologous sequences identified in each supergroup of eukaryotes. The shape of the dots represents the data corresponding to individual SF3b proteins (SF3b1- circle; SF3b2- triangle; SF3b3- square; SF3b4- cross; SF3b14b- pentagon; SF3b5- diamond; SF3b6- plus). In the x-axis label, microbial eukaryotes and higher-order eukaryotes are grouped by brown and green background colors, respectively, highlighting extensive coverage of species from distant lineages.

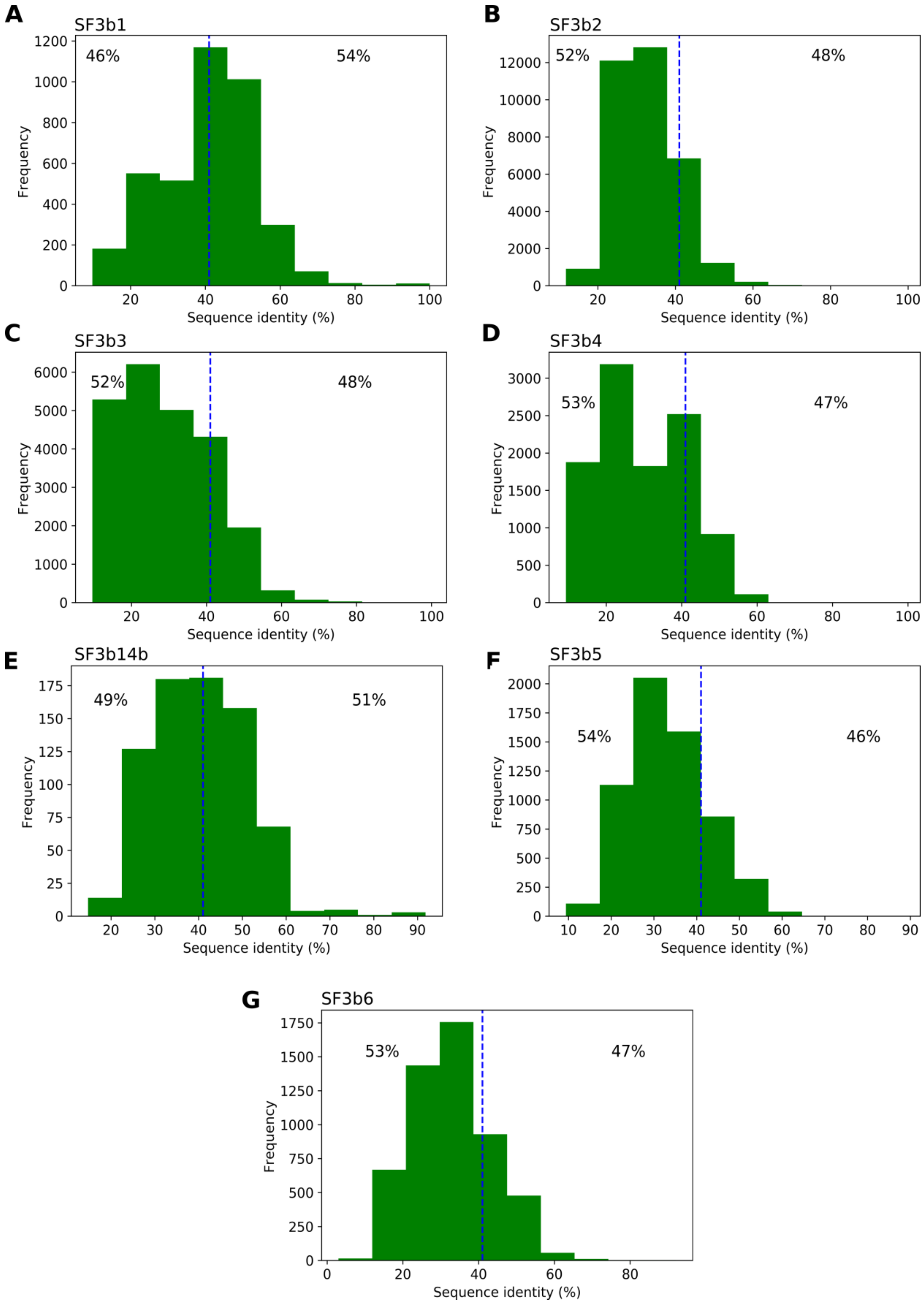

Figure S2. The distribution of pair-wise sequence identities among SF3b homologs.

Shown in A)-G) are the distributions of sequence identities between all-*vs*-all pairs of SF3b1,

SF3b2, SF3b3, SF3b4, SF3b14b, SF3b5 and SF3b6 homologs, respectively, considered for our sequence conservation analysis. Given in percentages within the plot are the percentage of homologous pairs sharing sequence identity below (left) and above (right) the average pair-wise sequence identity value of the distribution (vertical dashed blue line).

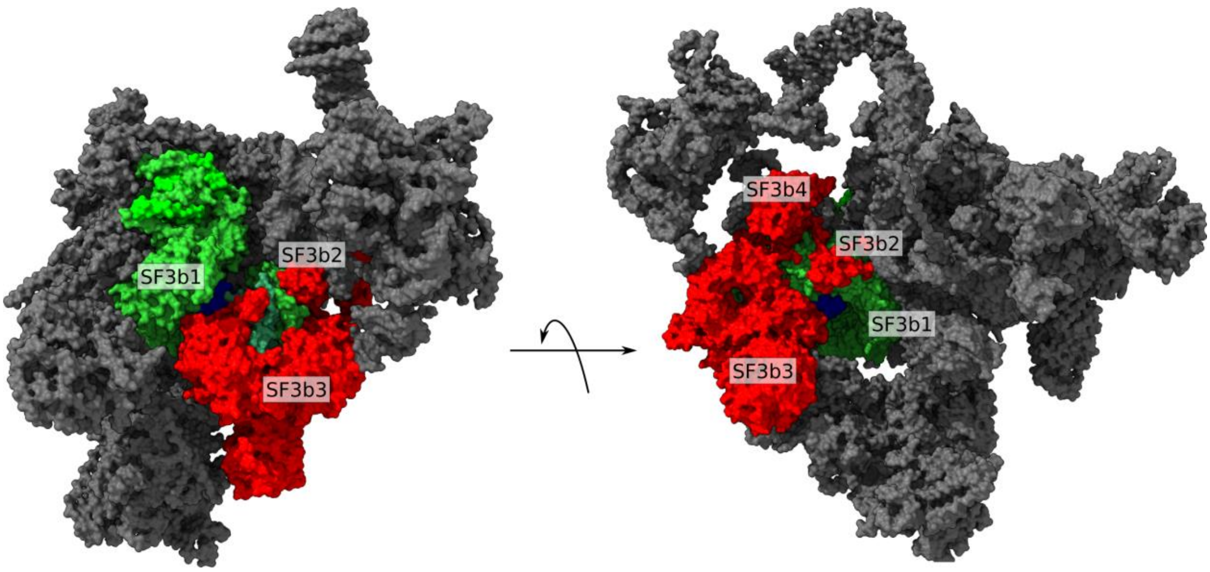

Figure S3. SF3b complex in the spliceosome. Shown are the cartoon representation of the SF3b complex in the context of the spliceosome assembly (grey, PDB code: 5Z58) (Zhang et al., 2018). SF3b components are colored based on their average JSD score. It is noted that SF3b1 is involved in many protein-protein interactions with other spliceosomal proteins than the remaining peripheral SF3b components *viz.* SF3b2, SF3b3 and SF3b4. The figure was generated using ChimeraX (Pettersen et al., 2021).

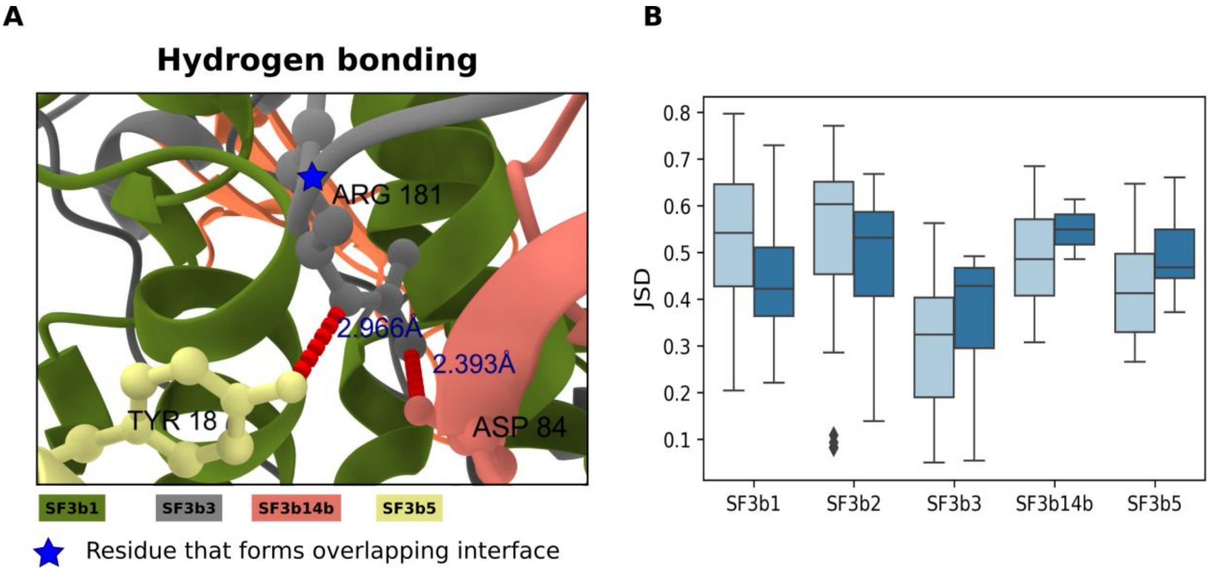

Figure S4. Residue conservation in the overlapping and non-overlapping interface regions. A) Shown as a cartoon is an example of bifurcated interactions formed by Arg181 in the yeast homolog of SF3b3 (indicated by a blue star). It interacts with SF3b14b and SF3b5 components via two hydrogen bonds and forms an overlapping interface. B) Box plot indicating the distribution of conservation score (JSD) for residues in the overlapping interface (dark blue) and the non-overlapping interface (light blue) in five SF3b components.

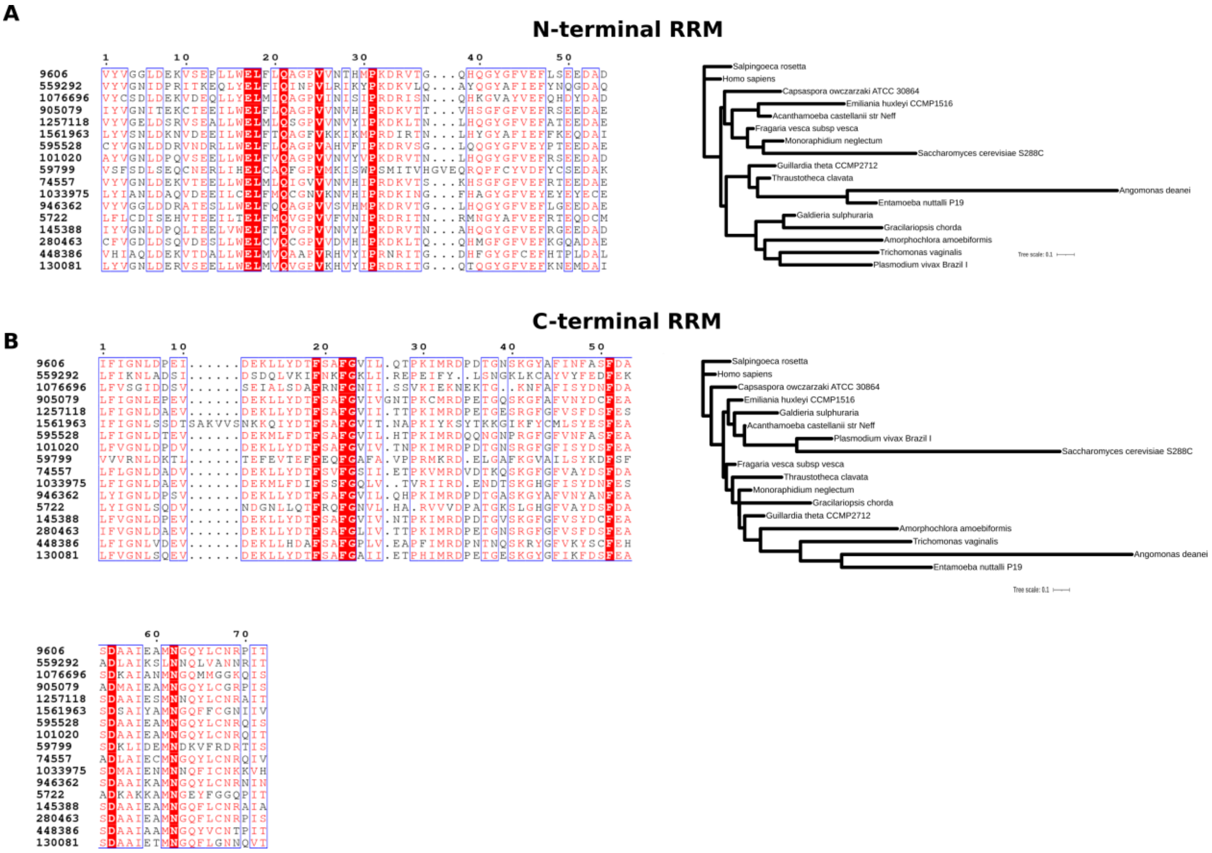

Figure S5. Sequence alignments and phylogenetic trees of RRM domains in SF3b4. A) Shown are the multiple sequence alignment of N-terminal RRM and corresponding maximum-likelihood method estimated phylogenetic tree in the left and right panel, respectively. B) Similar to (A) but for C-terminal RRM. The alignment figure was generated using ESPript tool (Robert and Gouet, 2014). The red background highlighted positions that are absolutely conserved sites while the blue border highlighted blocks indicate well conserved regions. The phylogenetic tree figures were generated using iTol tool (Letunic and Bork, 2021)

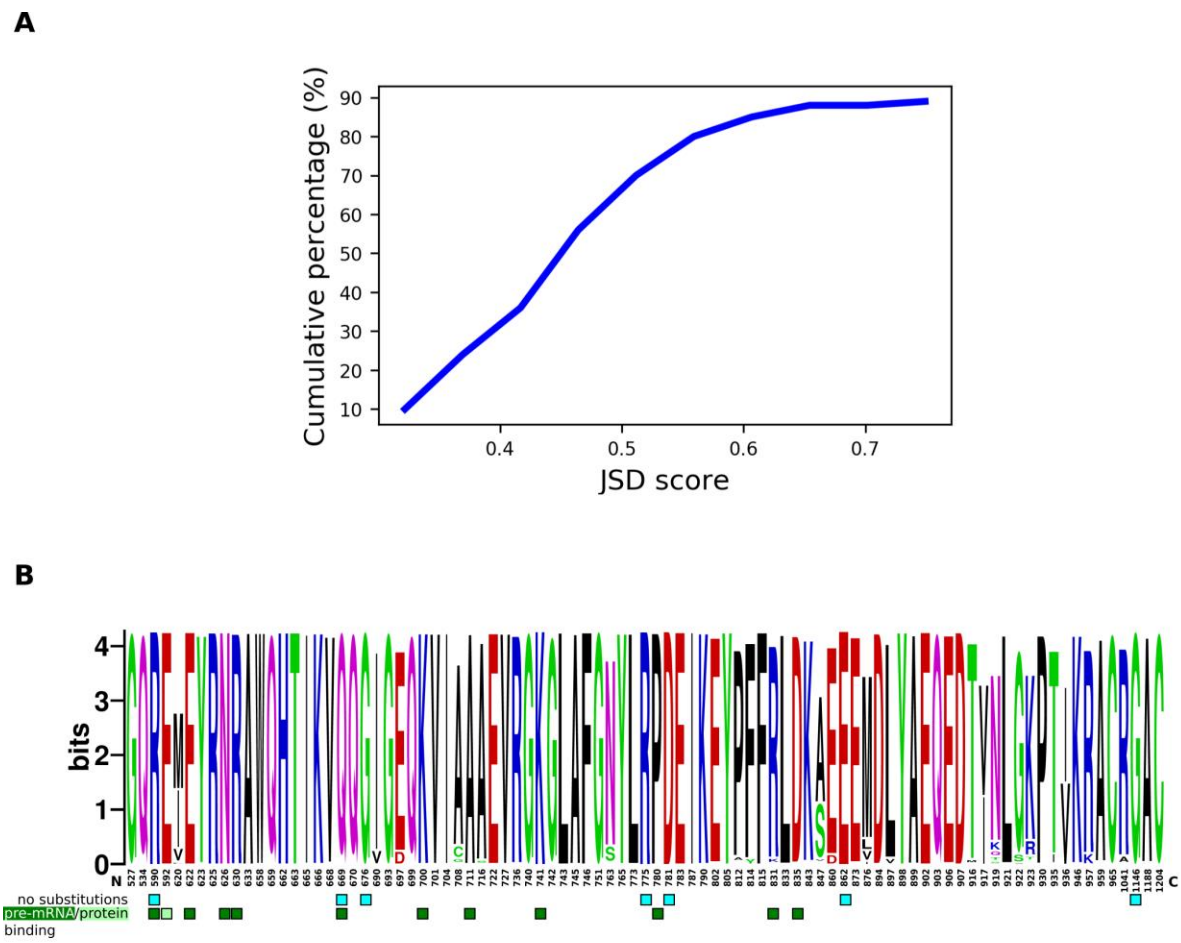

Figure S6. The conservation of cancer mutation sites in the SF3b1 homologs of metazoans. A) Cumulative frequency of JSD score of 89 cancer mutation sites in the SF3b1. The plot shows that more than 70% of sites have JSD score above 0.4. B) Shown is the sequence logo representing residue conservation in the form of bits score for 89 cancer mutation sites in SF3b1 across metazoans. The figure was generated from the sequence alignment of 490 metazoan SF3b1 homologs using WebLogo server (Crooks et al., 2004). Interface residues for pre-mRNA interactions (dark green) and SF3b14b interactions (light green) are highlighted by square boxes at the bottom. Cyan boxes mark residue positions that show no substitutions in metazoans.

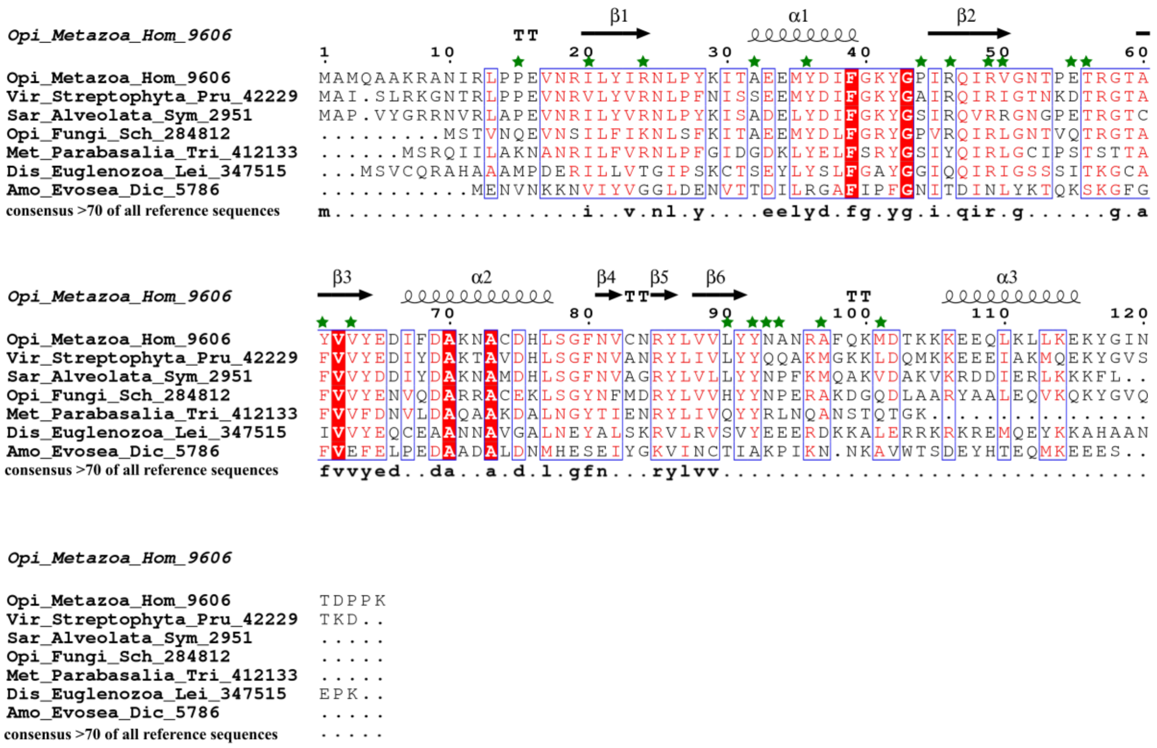

Figure S7. Sequence alignment of SF3b6. Sequence alignment of representative homologs of SF3b6. The alignment was visualized using the ESPript tool (Robert and Gouet, 2014). The color codes are same as the Figure S5. The green star symbol highlights the interface residues for SF3b1. The consensus sequence from the complete alignment comprising of 104 SF3b6 homologs covering all members of 60% clustered set is shown at the bottom of the alignment. Sequence name is given as taxonomy ID with the customized ID representing its taxonomy lineage (3-letter code of supergroup_kingdom_3-letter code of genus). The details of customized IDs are available in Supplementary Table S1.

References

Crooks, G. E., Hon, G., Chandonia, J. M., and Brenner, S. E. (2004). WebLogo: A sequence logo generator. Genome Research 14, 1188–1190. doi:10.1101/gr.849004.

Goldman, N., and Yang, Z. (1994). A codon-based model of nucleotide substitution for protein-coding DNA sequences. Molecular biology and evolution 11, 725–736. doi:10.1093/OXFORDJOURNALS.MOLBEV.A040153.

Kalyaanamoorthy, S., Minh, B. Q., Wong, T. K. F., von Haeseler, A., and Jermiin, L. S. (2017). ModelFinder: Fast model selection for accurate phylogenetic estimates. Nature Methods 14, 587–589. doi:10.1038/nmeth.4285.

Larsson, A. (2014). AliView: A fast and lightweight alignment viewer and editor for large datasets. Bioinformatics 30, 3276–3278. doi:10.1093/bioinformatics/btu531.

Letunic, I., and Bork, P. (2021). Interactive Tree Of Life (iTOL) v5: an online tool for phylogenetic tree display and annotation. Nucleic Acids Research 49, W293–W296. doi:10.1093/NAR/GKAB301.

Nguyen, L. T., Schmidt, H. A., von Haeseler, A., and Minh, B. Q. (2015). IQ-TREE: A fast and effective stochastic algorithm for estimating maximum-likelihood phylogenies. Molecular Biology and Evolution 32, 268–274. doi:10.1093/molbev/msu300.

Nielsen, R., and Yang, Z. (1998). Likelihood Models for Detecting Positively Selected Amino Acid Sites and Applications to the HIV-1 Envelope Gene. Genetics 148, 929–936. doi:10.1093/GENETICS/148.3.929.

Pettersen, E. F., Goddard, T. D., Huang, C. C., Meng, E. C., Couch, G. S., Croll, T. I., et al. (2021). UCSF ChimeraX: Structure visualization for researchers, educators, and developers. Protein Science 30, 70–82. doi:10.1002/pro.3943.

Robert, X., and Gouet, P. (2014). Deciphering key features in protein structures with the new ENDscript server. Nucleic Acids Research 42, W320-4. doi:10.1093/nar/gku316.

Rozewicki, J., Li, S., Amada, K. M., Standley, D. M., and Katoh, K. (2019). MAFFT-DASH: integrated protein sequence and structural alignment. Nucleic Acids Research 47, W5–W10. doi:10.1093/NAR/GKZ342.

Suyama, M., Torrents, D., and Bork, P. (2006). PAL2NAL: robust conversion of protein sequence alignments into the corresponding codon alignments. Nucleic Acids Research 34, W609–W612. doi:10.1093/nar/gkl315.

Yang, Z. (1997). PAML: a program package for phylogenetic analysis by maximum likelihood. Bioinformatics 13, 555–556. doi:10.1093/BIOINFORMATICS/13.5.555.

Yang, Z., and Nielsen, R. (1998). Synonymous and nonsynonymous rate variation in nuclear genes of mammals. Journal of Molecular Evolution 1998 46:4 46, 409–418. doi:10.1007/PL00006320.

Yang, Z., Nielsen, R., Goldman, N., and Pedersen, A. M. K. (2000). Codon-substitution models for heterogeneous selection pressure at amino acid sites. Genetics 155, 431–449. doi:10.1093/GENETICS/155.1.431.

Yang, Z., Wong, W. S. W., and Nielsen, R. (2005). Bayes Empirical Bayes Inference of Amino Acid Sites Under Positive Selection. Molecular Biology and Evolution 22, 1107–1118. doi:10.1093/MOLBEV/MSI097.

Zhang, X., Yan, C., Zhan, X., Li, L., Lei, J., and Shi, Y. (2018). Structure of the human activated spliceosome in three conformational states. Cell Research 28, 307–322. doi:10.1038/cr.2018.14.
